# Decoding the PITX2-controlled genetic network in atrial fibrillation

**DOI:** 10.1172/jci.insight.158895

**Published:** 2022-06-08

**Authors:** Jeffrey D. Steimle, Francisco J. Grisanti Canozo, Minjun Park, Zachary A. Kadow, Md. Abul Hassan Samee, James F. Martin

**Affiliations:** 1 Department of Integrative Physiology,; 2 Program in Developmental Biology, and; 3 Medical Scientist Training Program, Baylor College of Medicine, Houston, Texas, USA.; 4 Texas Heart Institute, Houston, Texas, USA.; 5 Center for Organ Repair and Renewal, Baylor College of Medicine, Houston, Texas, USA.

**Keywords:** Cardiology, Arrhythmias, Epigenetics, Transcription

## Abstract

Atrial fibrillation (AF), the most common sustained cardiac arrhythmia and a major risk factor for stroke, often arises through ectopic electrical impulses derived from the pulmonary veins (PVs). Sequence variants in enhancers controlling expression of the transcription factor *PITX2*, which is expressed in the cardiomyocytes (CMs) of the PV and left atrium (LA), have been implicated in AF predisposition. Single nuclei multiomic profiling of RNA and analysis of chromatin accessibility combined with spectral clustering uncovered distinct PV- and LA-enriched CM cell states. *Pitx2*-mutant PV and LA CMs exhibited gene expression changes consistent with cardiac dysfunction through cell type–distinct, PITX2-directed, cis-regulatory grammars controlling target gene expression. The perturbed network targets in each CM were enriched in distinct human AF predisposition genes, suggesting combinatorial risk for AF genesis. Our data further reveal that PV and LA *Pitx2*-mutant CMs signal to endothelial and endocardial cells through BMP10 signaling with pathogenic potential. This work provides a multiomic framework for interrogating the basis of AF predisposition in the PVs of humans.

## Introduction

Atrial fibrillation (AF) has a 25% lifetime risk and accounts for one-third of all cardiovascular diseases (1, 2). Debilitating complications linked to AF include stroke and heart failure, making early diagnosis, accurate prognosis, and effective treatment of the utmost importance (3). AF is characterized by a constant, disorganized atrial activity (2), which results most commonly from ectopic electrical impulses originating from pulmonary veins (PVs), which trigger left atrium (LA) depolarization competing with sinus rhythm (4). Because of the importance of the PV in AF pathophysiology, and because the PV is poorly understood, we performed a robust molecular interrogation of the PV alongside the better understood LA (5, 6).

AF risk is linked to common single nucleotide polymorphisms (SNPs) at dozens of loci (7, 8), including the noncoding AF-associated region (AFAR) at the 4q25 locus in enhancers controlling *PITX2* expression (9), which encodes the developmentally critical, paired-like homeodomain transcription factor (TF), PITX2. Our recent work demonstrated that the AFAR contains multiple cis-regulatory element (CRE) signatures, and makes 3-dimensional contact with the *Pitx2* promoter in mouse LA cardiomyocytes (CMs), and has enhancer activity (9). PITX2, required for the development of the PV and LA, is expressed in the CMs of the adult LA, where it has been implicated in AF pathophysiology in both patients and animal models (9–14). Deletion of the AFAR in mice results in decreased *Pitx2* expression and increases in inflammation, *Bmp10* expression, and AF susceptibility (9), similar to human patients with AFAR SNPs (15). Although these previous studies suggested a molecular role for *Pitx2* in adult LA myocardium in AF susceptibility (9, 13, 16), a comprehensive understanding of the downstream transcriptional and epigenetic role of *Pitx2* is limited in the LA and unexplored in the PV (6, 17).

PITX2, as do all TFs, functions cooperatively and antagonistically through interaction with other TFs to regulate transcription at CREs in CMs. For example, PITX2 interacts with NRF2 to cooperatively regulate oxidative stress response in CMs (17–19), while antagonistically regulating TBX5-dependent CREs in LA CMs, indicating that PITX2 acts in various cis-regulatory grammars to regulate transcription in adult CMs (20, 21). A comprehensive understanding of how PITX2 acts on TF grammars in PV CMs is unknown.

To investigate *Pitx2* function in the adult PV and LA, we performed single nuclei RNA sequencing (snRNA-Seq) and single nuclei assay for transposase-accessible chromatin sequencing (snATAC-Seq) on nuclei from PV and LA from control and *Pitx2*-mutant mice. We found 3 CM populations, 2 of which are resident in PV, including 1 with an expression signature of automaticity. *Pitx2*-mutant CMs exhibited differentially expressed genes (DEGs) that are associated with accessible PITX2 binding motifs. Moreover, these PITX2 binding motifs are associated with distinct regulatory grammars in different CM populations. These downstream DEGs are enriched for other AF predisposition genes identified in humans. Last, mutant CMs of the PV and LA signal to endothelial and endocardial cells through BMP10. Together, our work provides a robust set of transcriptional and epigenetic data sets that will provide a platform for interrogating the LA and PV in AF predisposition.

## Results

### Single nuclei profiling of Pitx2-mutant LA and PV.

**Figure 1:**
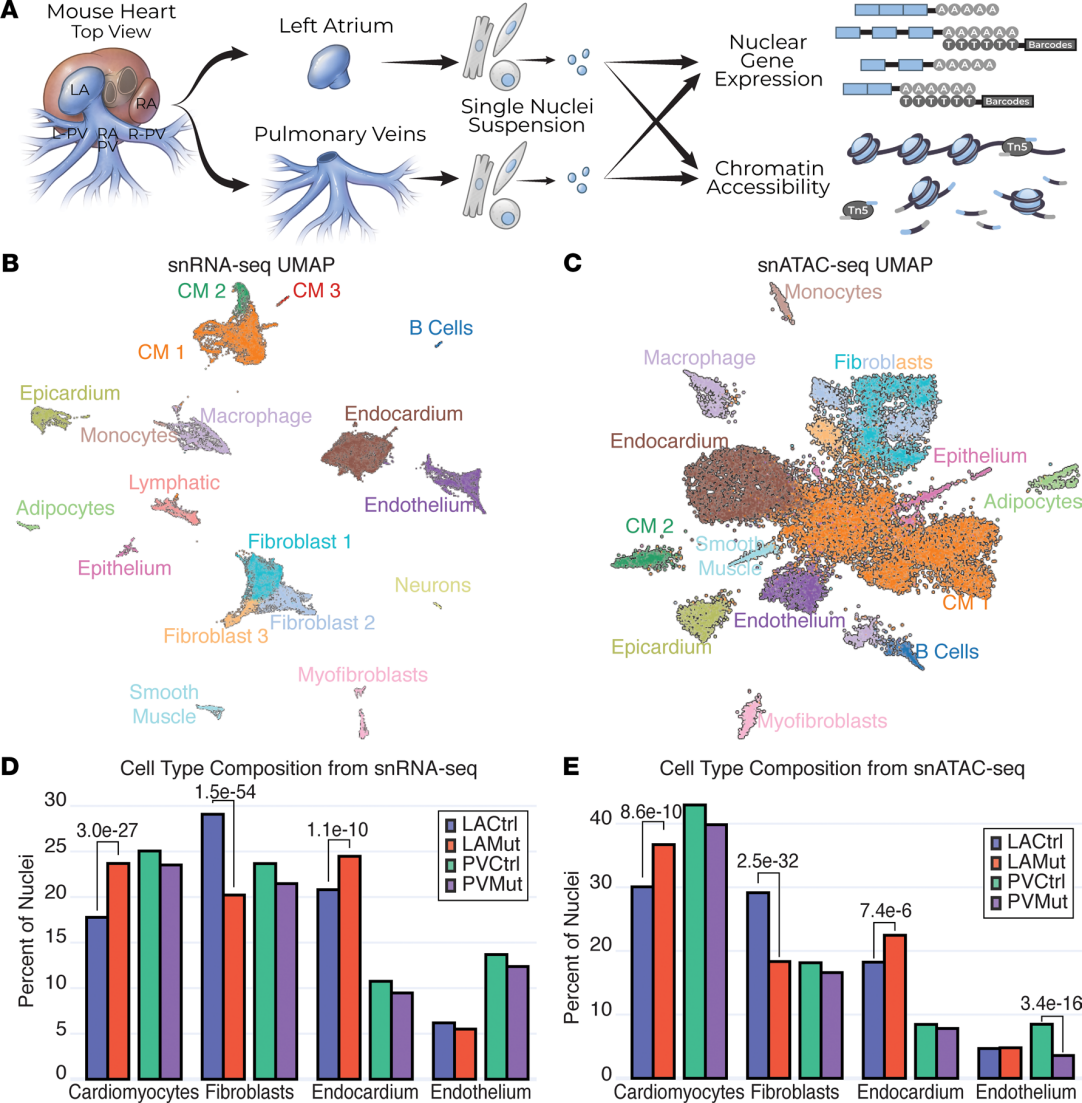
Single nuclei profiling of the pulmonary vein and left atrium. (**A**) Experimental outline used to profile the transcriptome and chromatin accessibility of single nuclei of the left atrium (LA) and pulmonary vein (PV) from pools of 6- to 8-month-old *Pitx2* control (Ctrl: *Pitx2^fl/+^*) and mutant (Mut: *MCK-cre* *Pitx2^fl/–^*) mice. (**B**) Uniform manifold approximation and projection (UMAP) representation of all filtered nuclei identified by single nuclei RNA-sequencing (snRNA-Seq) and color-coded and labeled in clusters. (**C**) UMAP representation of single nuclei assay for transposase-accessible chromatin using sequencing (snATAC-Seq) with colors and labels lifted from the snRNA-Seq (in **B**). (**D**) Percentage of total nuclei per sample from the 4 major clusters identified in the snRNA-Seq data set. (**E**) Percentage of total nuclei per sample identified in the snATAC-Seq data set. Adjusted *P* value (FDR) of significant comparisons (FDR < 1 × 10^–5^) between LA or PV control and mutant samples are presented.

*Pitx2* is expressed in and required for the development of the PV and left atrial myocardium (11–13). Moreover, *Pitx2* expression persists into the adult, albeit at reduced levels (13). To investigate *Pitx2* function in adult PV and LA, we used the *MCK-cre* *Pitx2*^fl/–^ (referred to as *Pitx2* mutant, Methods) mouse model, which we have shown has sinus node dysfunction and atrial conduction defects, 2 AF-predisposing conditions in humans (22, 23). We microdissected and isolated nuclei from PV and LA of *Pitx2* controls (*Pitx2*^fl/+^) and *Pitx2* mutants (Figure 1A) and performed snRNA-Seq and snATAC-Seq.

Following quality control and removal of low-quality nuclei by CellRanger and CellBender (24) (Methods and Supplemental Figure 1; supplemental material available online with this article; https://doi.org/10.1172/jci.insight.158895DS1), we performed dimension reduction and integrated cluster analysis on 41,940 nuclei across the 4 samples using Seurat (25–27) (Figure 1B). We identified 18 clusters representing 14 cell types, including 3 clusters of CMs, based on marker gene expression (Figure 1B, Supplemental Figure 2, and Supplemental Table 1). Likewise, for snATAC-Seq, we performed dimension reduction and integrated cluster analysis on 18,654 nuclei across 4 samples following quality control and removal of low-quality nuclei using Signac (26, 28) (Methods, Figure 1C, and Supplemental Figure 1). Cluster cell type annotations for snATAC-Seq nuclei, derived from snRNA-Seq, revealed 15 distinct clusters based on chromatin accessibility (Methods; Figure 1, B and C; and Supplemental Figure 3).

We asked whether the composition of the 4 major PV and LA cell types, making up 67%–82% of all nuclei per sample, had significant differences between genotypes. Using a stringent cutoff (FDR < 1 × 10^–5^), we found that in both the snRNA-Seq and snATAC-Seq data sets, CMs (snRNA-Seq: χ^2^ = 119.7 and FDR = 3.0 × 10^–27^; snATAC-Seq: χ^2^ = 39.5 and FDR = 8.6 × 10^–10^) and endocardium (EndoC) (snRNA-Seq: χ^2^ = 43.5 and FDR = 1.1 × 10^–10^; snATAC-Seq: χ^2^ = 21.4 and FDR = 7.4 × 10^–6^) represented a greater proportion of nuclei in *Pitx2*-mutant LA (Figure 1, D and E). There was an approximate gain of 6% in CMs and 4% in EndoC for both data types in the *Pitx2*-mutant LA. Additionally, *Pitx2*-mutant LA fibroblasts (FBs) represented a reduced proportion of nuclei overall (snRNA-Seq: 9% less, χ^2^ = 246.1, and FDR = 1.5 × 10^–54^; snATAC-Seq: 11% less, χ^2^ = 144.3, and FDR = 2.5 × 10^–32^). In contrast, the PV had no cellular compositional changes between control and *Pitx2*-mutant samples apart from PV endothelium (Endo) from snATAC-Seq data, which had a reduced proportion of nuclei in *Pitx2*-mutant condition (5% less, χ^2^ = 69.3, and FDR = 3.4 × 10^–16^; Figure 1, D and E). Together, our data suggest that *Pitx2* regulates LA cellular composition, consistent with compositional differences observed in the LA and right atrium of human snRNA-Seq (29); however, we did not detect strong differences in PVs (Figure 1, D and E).

### Distinct CM subpopulations in PVs.

**Figure 2:**
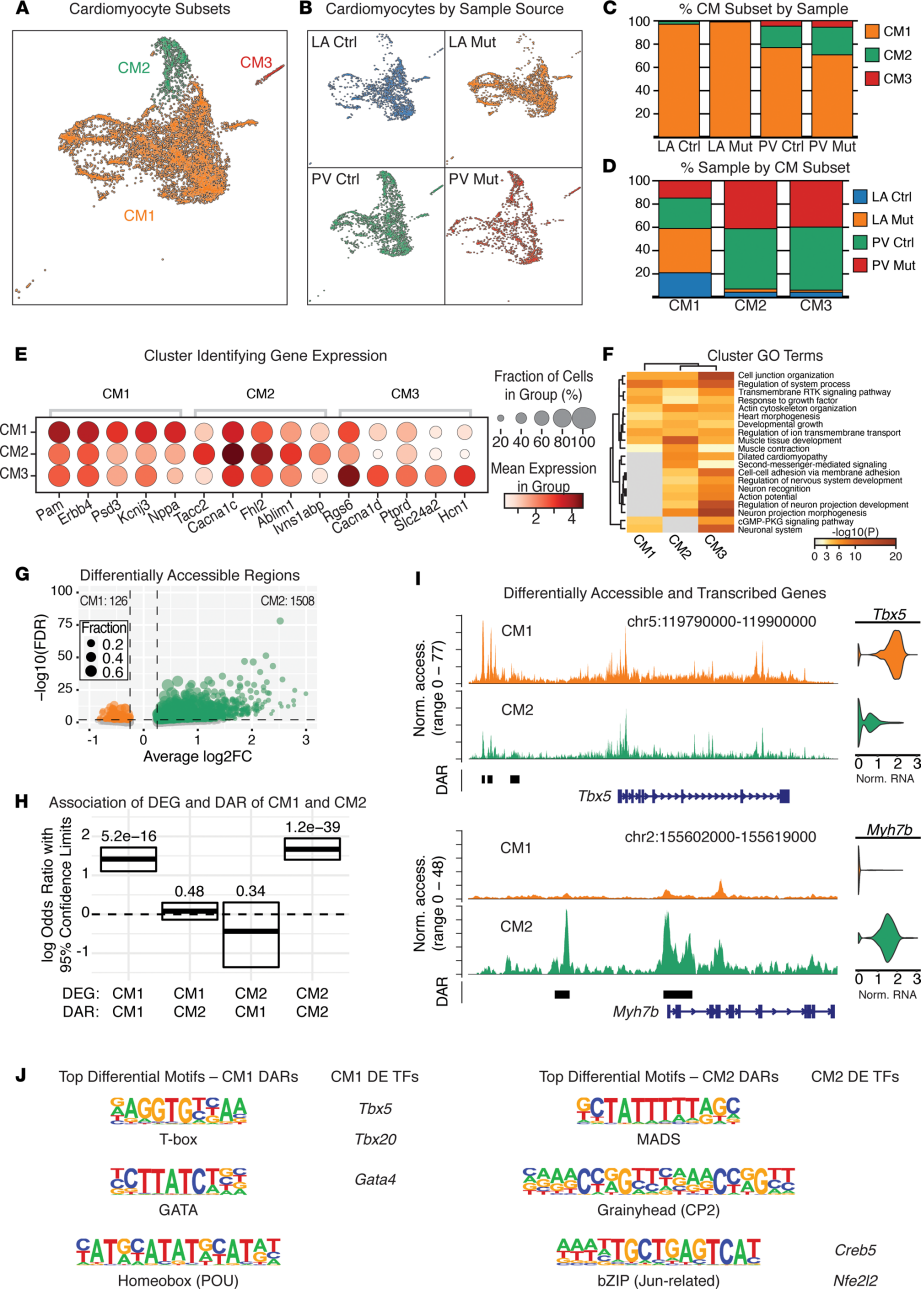
Identification of PV-enriched cardiomyocyte populations. (**A**) Uniform manifold approximation and projection (UMAP) representation of the cardiomyocyte (CM) subsets alone. (**B**) UMAP of CM subsets separated and colored by sample source. (**C**) The percentage of each CM subset in each sample. (**D**) The percentage CM subset from a given sample source. (**E**) Top CM subset markers identified by multiple pairwise comparison. Full list of markers is in Supplemental Table 2. (**F**) Heatmap of top 20 parent Gene Ontology (GO) terms identified across the 3 CM subsets. Complete details and child terms can be found in Supplemental Table 3. (**G**) Volcano plot showing the distribution of differentially accessible regions (DARs) between CM1 and CM2 (Supplemental Table 4). (**H**) Odds ratio plot by Fisher’s exact test for the association between differentially expressed genes (DEGs) and DARs enriched in CM1 or CM2. Significance values represent the adjusted *P* value (FDR). (**I**) Genome browser views at *Tbx5* (top) and *Myh7b* (bottom). Pseudo-bulk ATAC signal plotted for CM1 and CM2 with DARs highlighted. On the right, violin plots representing normalized RNA expression. (**J**) Top 3 differential motifs identified for CM1 DARs and CM2 DARs alongside the list of any differentially expressed transcription factors (DE TFs) corresponding to each identified motif family.

Using iterative clustering, we identified 3 CM clusters in the snRNA-Seq data, namely CM1, CM2, and CM3 (Figure 2A). By separating samples by tissue source, PV versus LA, we found that both the control and *Pitx2*-mutant LA were predominantly composed of CM1 (Figure 2, B–D). In contrast, the PVs from both control and *Pitx2*-mutant samples were composed of a mixture of CM1 (75%), CM2 (20%), and CM3 (5%) (Figure 2, B–D).

We next performed pairwise differential gene expression testing between CM1, CM2, and CM3 (Supplemental Table 2). Among the genes with highest expression in CM1, we identified several atrial markers, including *Nppa* and *Kcnj3*, which are both associated with AF (30, 31) (Figure 2E). Genes enriched in CM2 included the L-type calcium channel *Cacna1c*, associated with sustained AF (32); *Ablim1*, which encodes an actin binding protein associated with left-right asymmetry and is expressed in the developing LA (33); *Ivnsabp1*, encoding an actin binding protein that functions in cytoskeleton stabilization (34); *Fhl2*, encoding a LIM-containing protein associated with adrenergic stress signaling (35); and *Tacc2*, a centrosomal protein (36). For genes enriched in CM3, we identified several ion channels associated with AF and cardiac automaticity, including *Hcn1*, *Cacna1d*, and *Slc24a2* (7, 37).

To further characterize CMs, we performed pathway analysis on the complete set of CM-enriched genes (Figure 2F) (38). CM3 exhibited many overlapping terms associated with neuronal, ion channel, and cell-cell adhesion and intercellular communication due to many expressed ion channel genes (Figure 2F and Supplemental Table 3). Intriguingly, CM3 also expressed genes associated with sinoatrial node cell development, specifically the transcription factors *Tbx3*, *Tbx18*, and *Shox2*, consistent with the hypothesis that CM3 has automaticity at homeostasis (Figure 2, E and F, and Supplemental Tables 2 and 3). CM2 exhibited several overlapping pathways associated with muscle cell development, muscle contraction, and cytoskeletal organization owing to its enrichment in several core components of the cardiac contractile machinery consistent with previous ultrastructural studies of PV CMs (39) (Figure 2F and Supplemental Table 3). In addition, CM2-enriched genes were uniquely associated with carbohydrate metabolism, e.g., *Gck*, *Ugp2*, and *Gys1* (Supplemental Tables 2 and 3). CM1-enriched genes represented pathways of cardiac contraction and ion channels (Figure 2F and Supplemental Table 3).

To test for differences in chromatin accessibility between CM populations, we identified differentially accessible regions (DARs) by comparing the snATAC-Seq of CM1 and CM2, since chromatin accessibility of CM1 and CM3 was indistinguishable (Supplemental Figure 3). We found 1634 DARs with 126 regions more accessible in CM1 and 1508 regions more accessible in CM2 (Figure 2G and Supplemental Table 4). Utilizing available Hi-C data to identify topologically associating domains (TADs) and inter-TAD spaces (40, 41), we linked DARs with transcriptional start sites (TSSs) present within the same TAD/inter-TAD. We identified 54 genes more highly expressed in CM1 that were associated with more accessible DARs (Fisher’s exact test, FET, *P* = 5.2 × 10^–16^; odds ratio, OR = 4.1) and 194 genes more highly expressed in CM2 associated with more accessible DARs (FET *P* = 1.2 × 10^–39^; OR = 5.3; Figure 2H and Supplemental Table 4). In CM1, we identified several AF-associated genes, namely, *Tbx5*, *Sox5*, *Slit3*, *Smad7*, and *Nppa*. CM2 expressed AF-associated genes *Efna5*, *Cpeb4*, *Myot*, *Pln*, *Rpl3l*, *Arhgap26*, *Thrb*, *Mapt*, and *Mypn* (Figure 2I and Supplemental Figure 4). Together, DARs of CM1 and CM2 identify a strong association between gene expression and chromatin accessibility.

Next, we performed motif enrichment analysis to identify the potential TFs driving the DARs in CM1 and CM2. For CM1, we found DARs were enriched for T-box, GATA, and POU homeodomain factors compared with CM2 DARs, while CM2 DARs were enriched for MADS, CP2 Grainyhead, and Jun-related bZIPs relative to CM1 (Figure 2J). Of interest, the DEG analysis between CM1 and CM2 identified several factors in these families that corresponded with the motif analysis, including *Tbx5*, *Tbx20*, and *Gata4* for CM1 and *Creb5* and *Nfe2l2* for CM2 (Figure 2J). In both cases, these factors are consistent with the Gene Ontology (GO) term analysis of all DEGs (Figure 2F and Supplemental Table 3). CM1 represents atrial-like CMs, for which TBX5 is a master regulator of identity (16, 42). On the other hand, CM2 has a signature of oxidative stress mediation and metabolic regulation, exemplified by *Nfe2l2*, encoding NRF2, a well-established regulator in multisystem disorders (43) and a cofactor of PITX2 in oxidative stress responses of the heart and eye (19, 44).

### Pitx2-dependent transcription of PV and LA CMs.

**Figure 3:**
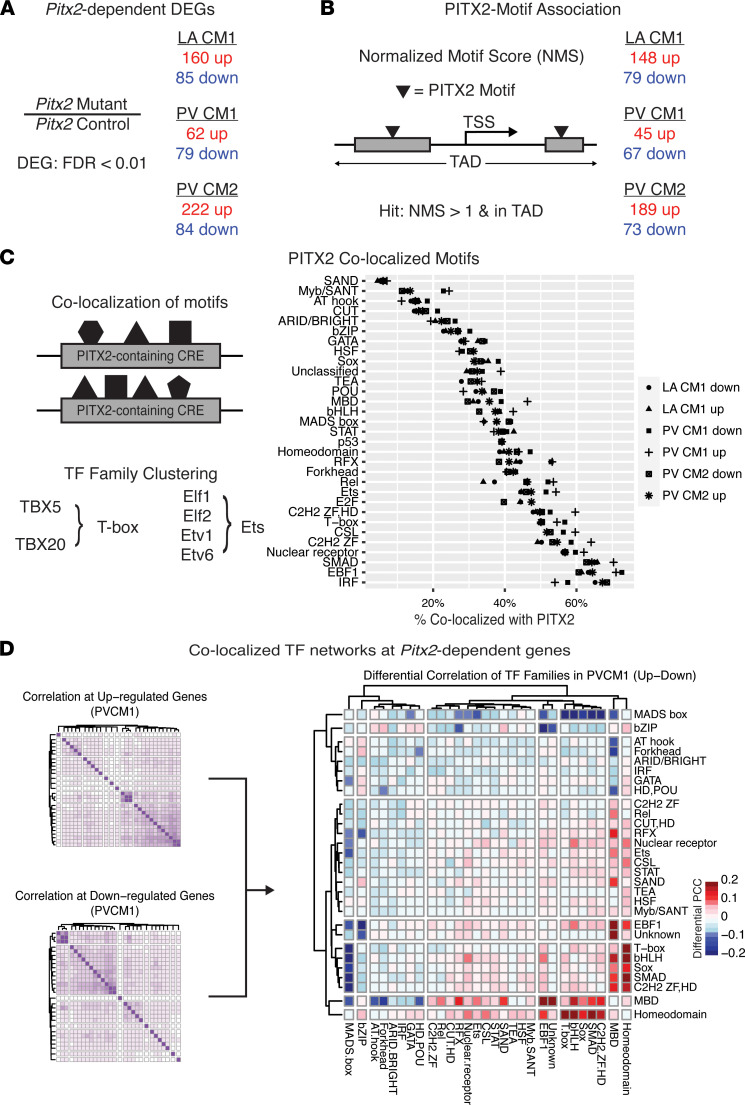
Systems biology approach to PITX2-dependent regulatory networks. (**A**) Quantification of differentially expressed genes (DEGs) identified by subset comparing controls and *Pitx2* mutants. Complete list including PV CM3 is in Supplemental Table 5. (**B**) Number of DEGs associated with a cis-regulatory element (CRE) with a PITX2 normalized motif score (NMS) > 1 for each subset. (**C**) The mean percentage of colocalized motifs by transcription factor (TF) family. Colocalization was defined as the occurrence of at least 1 motif at a PITX2-containing CRE (NMS > 1) associated with a DEG in the given comparison. Only expressed TFs in each cell type were considered. Complete breakdown for each expressed TF by comparison is located in Supplemental Figure 6. (**D**) Identification of differentially correlating TF family networks at PV CM1 by Pearson’s correlation coefficient. Detailed correlation heatmaps for PV CM1 along with LA CM1 and PV CM2 are located in Supplemental Figure 8.

To test PITX2-regulated gene expression in PVs and LA, we performed differential gene expression testing between controls and *Pitx2* mutants for each of the LA CM1, PV CM1, PV CM2, and PV CM3 populations (Figure 3A and Supplemental Table 5). Among the comparisons, we identified hundreds of up- and downregulated DEGs in the *Pitx2*-mutant populations (Figure 3A and Supplemental Table 5). While many DEGs were only detected in 1 comparison, there were several DEGs with shared differences; i.e., *Malat1*, *Mapk10*, *Sorbs2os*, and *Actn2* were downregulated across all 4 comparisons, while *Ccnd3*, *9530026P05Rik*, and *Ckm* were consistently upregulated (Supplemental Table 5). In addition, in *Pitx2* mutants, the CM1 populations of the PV and LA both showed a gain in *Bmp10* and *Etv1* and a loss in *Stat3*, *Nr4a3*, *Pdlim5*, and *Slc24a2*, among other genes with known roles in AF predisposition (Supplemental Table 6).

To test the association between PITX2 binding and transcriptional cis-regulation of DEGs, we first computed PITX2 normalized motif scores (NMSs), which are a function of chromatin accessibility and PITX2 motif occurrences weighted by length, at accessible regions (Methods). PITX2 footprinting using snATAC-Seq (45) was associated with sites of high PITX2 NMS (Supplemental Figure 5A). Using published TADs (40, 41), we next assigned each scored region to DEGs within the same TAD (Figure 3B, Methods). The vast majority (73%–93%) of DEGs from each of the LA CM1, PV CM1, and PV CM2 comparisons were associated with accessible regions containing PITX2 motifs. We refer to these regions as PITX2-containing CREs at DEGs (Supplemental Table 7). Comparing the PITX2-containing CREs with the VISTA (46) and ENCODE (47) databases revealed a significant enrichment of these sites as predicted or validated CREs in the heart (Supplemental Figure 5B). Together, these data support the conclusion that DEGs of the PV and LA are associated with PITX2 binding and transcriptional activity.

To further elucidate PITX2-mediated transcriptional cis-regulatory mechanisms, we focused on PITX2-containing CREs associated with genes whose expression was changed in *Pitx2* mutants compared to control (*Pitx2*-dependent genes). We hypothesized that identification of co-occurring motifs would uncover PITX2 cofactors, which would provide insight into the underlying differences in *Pitx2*-dependent gene expression of PV and LA CMs. We performed motif scanning on the union set of accessible chromatin in CM1 and CM2 and binned individually expressed TFs by TF family. Globally, many TF family motifs co-occurred with PITX2 at similar rates, irrespective of CM population or directionality of gene expression difference. Included among the TF families we uncovered are previously validated PITX2 cofactors including T-box, SMAD, and Forkhead families (Figure 3C and Supplemental Figure 6) (16, 48–50).

CREs are constrained in terms of the identity and the number of TF motifs that they contain, i.e., the TF grammar, an essential component of combinatorial TF activity directing transcription. Disruption to TF grammar is sufficient to disrupt transcriptional activity at target genes (21). In our analysis (Figure 3C), we focused on a one-to-one relationship between PITX2 and other motifs. We hypothesized that *Pitx2*-dependent gene expression depends on cis-regulatory grammar within PITX2-containing CREs and that differentially expressed TFs play a combinatorial role in cis-regulation of *Pitx2* target genes. To address this, we correlated previously identified motif families in PITX2-containing CREs at up- and downregulated genes in each of the PV and LA CMs (Figure 3C and Supplemental Figure 6). Using these correlation matrices, we performed spectral clustering to generate interaction networks of PITX2 co-occurring motif families (Supplemental Figure 7). Spectral clustering is a method of clustering after dimensionality reduction to identify groups of nodes based on their connecting edges, i.e., clusters of TF families based on pairwise overlap fixed on occurrences with a PITX2 motif. Through this method, we identified several consistent interactions in PITX2-containing CREs at both up- and downregulated DEGs, e.g., bHLH-Sox-SMAD or C2H2 ZF-IRF (Supplemental Figure 7). Second, differentially expressed TFs were often strong nodes within each network, e.g., *Tbx20* (T-box) at PV CM1 upregulated genes or *Tcf7l2* (Sox) at PV CM2 up- and downregulated genes (Supplemental Figure 7).

Last, we wanted to identify the strongest differences between the CRE grammar dictating up- and downregulated genes, including whether PITX2 may be a direct driver. To do this, we contrasted the correlation matrices of motifs at all accessible chromatin sites irrespective of PITX2 NMS associated with up- and downregulated genes for each of LA CM1, PV CM1, and PV CM2 (Figure 3D and Supplemental Figure 8). The most striking difference was found in the PV CM1, where the T-box, bHLH, Sox, and SMAD motifs were more strongly associated with the PITX2 motif (homeodomain family) and MBD family at upregulated genes, while those same families of motifs were more strongly associated with the MADS box motifs at downregulated genes (Figure 3D). Additionally, for PV CM2, SMAD and bHLH families demonstrated a strong interaction at upregulated genes (Supplemental Figure 8), and the SMAD/E-box factor *Nfia* and bHLH factor *Tcf12* were both upregulated in the PV CM2 population from *Pitx2* mutants (Supplemental Figure 7), suggesting that gain of these factors in PV CM2 may be driving the upregulated DEGs. Altogether, our data suggest that the *Pitx2*-dependent gene expression changes observed in each of the LA and PV CM subsets may be governed by different sets of TF interaction networks and that these regulatory grammars are responding differently in the different populations and tissues.

### Pitx2-dependent gene expression associates with AF SNPs and gene targets.

**Figure 4:**
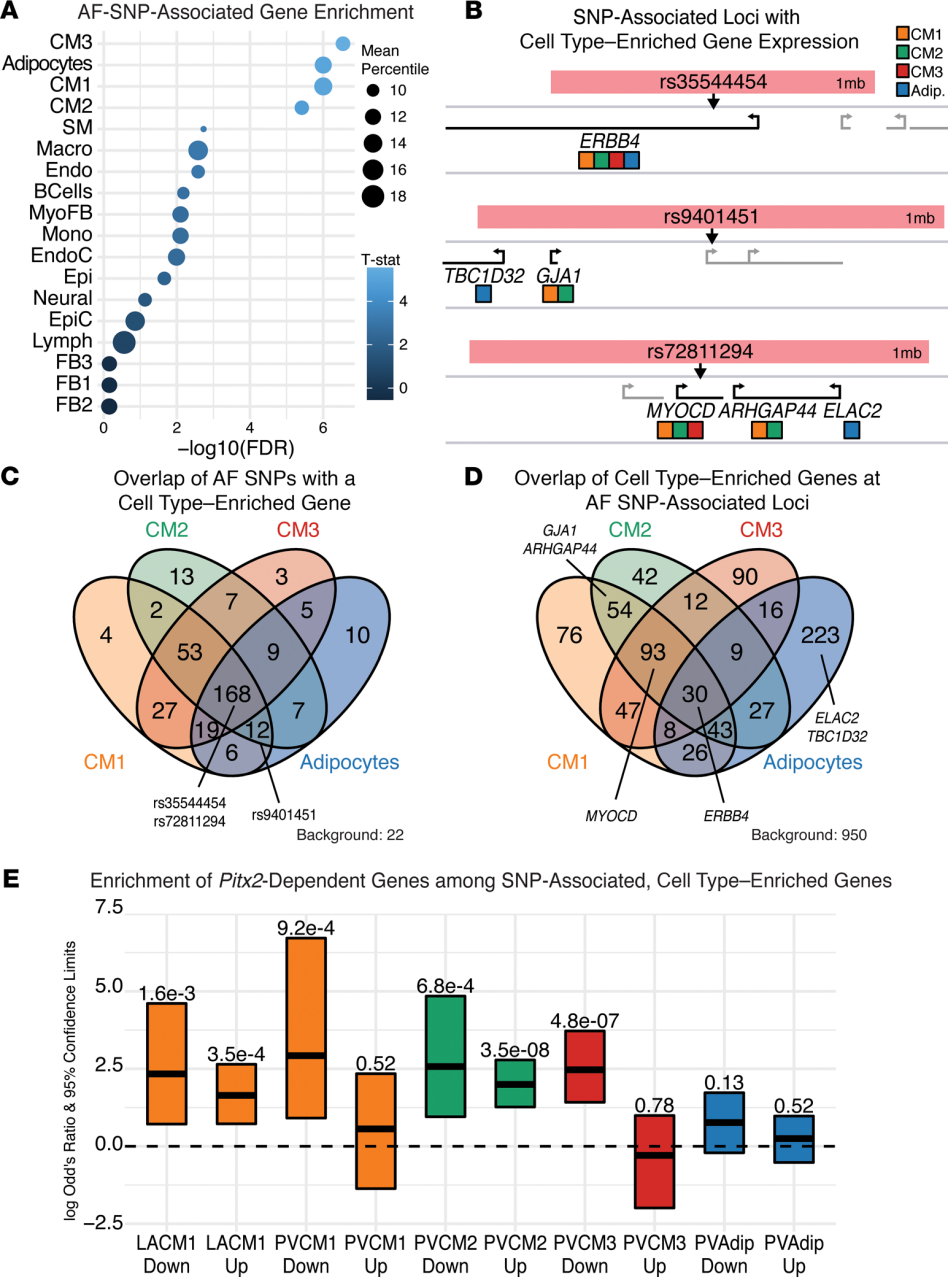
Human AF GWAS enrichment in perturbed PITX2 networks. (**A**) Enrichment scores of normalized and binned gene expression by cell subset for AF GWAS–associated genes. (**B**) Example AF-SNP loci (arrows) with coding genes within the 1 Mb window demonstrating top 20th percentile enrichment in CM1 (orange), CM2 (green), CM3 (red), or adipocytes (blue. (**C**) Venn diagram comparing the overlapping AF SNPs associated with at least 1 highly cell type–enriched gene (20th percentile). A total of 22 SNPs were not associated with any of the 4 cell types by our method. (**D**) Venn diagram comparing the 20th percentile cell type–enriched genes underlying the SNPs in **C**. (**E**) Enrichment scores by Fisher’s exact test for the overlap of previously identified *Pitx2*-dependent genes in CMs and adipocytes with the SNP-associated, cell type–enriched genes identified in **D**. Adjusted *P* value (FDR) for each enrichment test shown.

As the variation detected by human GWAS affects CREs and gene expression (17, 51), overlaying mouse snRNA-Seq data with GWAS can illuminate cell types and genetic pathways important for disease susceptibility (52). To understand the effects perturbed PITX2-dependent genes have on AF risk, we compared our snRNA-Seq data from mice with the GWAS catalog of reported AF SNPs (53). We identified all human genes within ±500 kb of the reported SNP (54) with homology in the mouse (55–58), as synteny is well conserved at this distance (59), and defined these as SNP-associated genes. We compared the relative gene expression of the SNP-associated genes (Methods) between our 18 cell types (Figure 1) and asked which clusters had a higher SNP-associated gene expression relative to non-SNP-associated gene expression. Using this approach, we identified SNP-associated enrichment for CM1, CM2, and CM3 (Figure 4A), consistent with recent reports from human snRNA-Seq data sets (29, 60). We also identified the adipocyte population (Figure 4A). While not previously associated with AF GWAS, a biologically related trait, PR interval, was associated with adipocyte gene expression in humans (29). Interestingly, this analysis identified no link between AF GWAS and the fibroblast populations, suggesting genetic predisposition is decoupled from FBs whose role in the pathogenesis of AF may be secondary to changes in the CMs.

Next, we asked which SNPs and genes were identified by each population (Figure 4B). Using only SNP-associated genes binned in the top 20th percentile for each population (Methods), we examined the overlap of SNPs between the populations (Figure 4C). First, we found that almost all the AF SNPs (91%) are represented by at least 1 gene in any CM population. Furthermore, 60% of the AF SNPs are associated with a top 20th percentile gene in all 3 CM populations. Since there was a strong overlap between the SNPs identified, we asked whether the associated genes were the same (Figure 4D). Once again, while the CM populations did share several genes, many of the subsets demonstrated uniquely identified genes (Figure 4D). While the adipocytes identified many of the same SNPs as the CMs (Figure 4C), almost none of the genes identified were shared with the CMs (Figure 4D) and were strongly enriched with terms associated with adipocytes (Supplemental Figure 9). This adipocyte observation is certainly of interest (61) but may ultimately prove a red herring (62).

Loss-of-function SNPs associated with *PITX2* are known to be one of the strongest predictors of AF and AF recurrence (63–65), and we have demonstrated previously that decreased *Pitx2* expression predisposes mice to AF (9, 13) with PITX2 transcriptional networks disrupted (Figure 3). To identify if the disrupted networks are associated with other GWAS candidate genes, we compared *Pitx2*-dependent gene expression changes with the SNP-associated genes enriched among the top 20th percentile identified previously (Figure 4D). Genes downregulated in LA CM1, PV CM1, PV CM2, and PV CM3 from *Pitx2* mutants were all significantly enriched among the AF GWAS–associated genes (Figure 4E). Furthermore, genes upregulated in LA CM1 and PV CM2 were also enriched among the 20th percentile genes (Figure 4E). Consistent with our observation that PITX2 networks are different in the various CM populations, the DEGs overlapping SNP-associated genes were different, with a few notable exceptions like *Etv1*, *Erbb4*, *Slc24a2*, and *Lpl* (Supplemental Figure 9).

### BMP10 mediates cell non-autonomous effects of Pitx2 in the EndoC and Endo.

**Figure 5:**
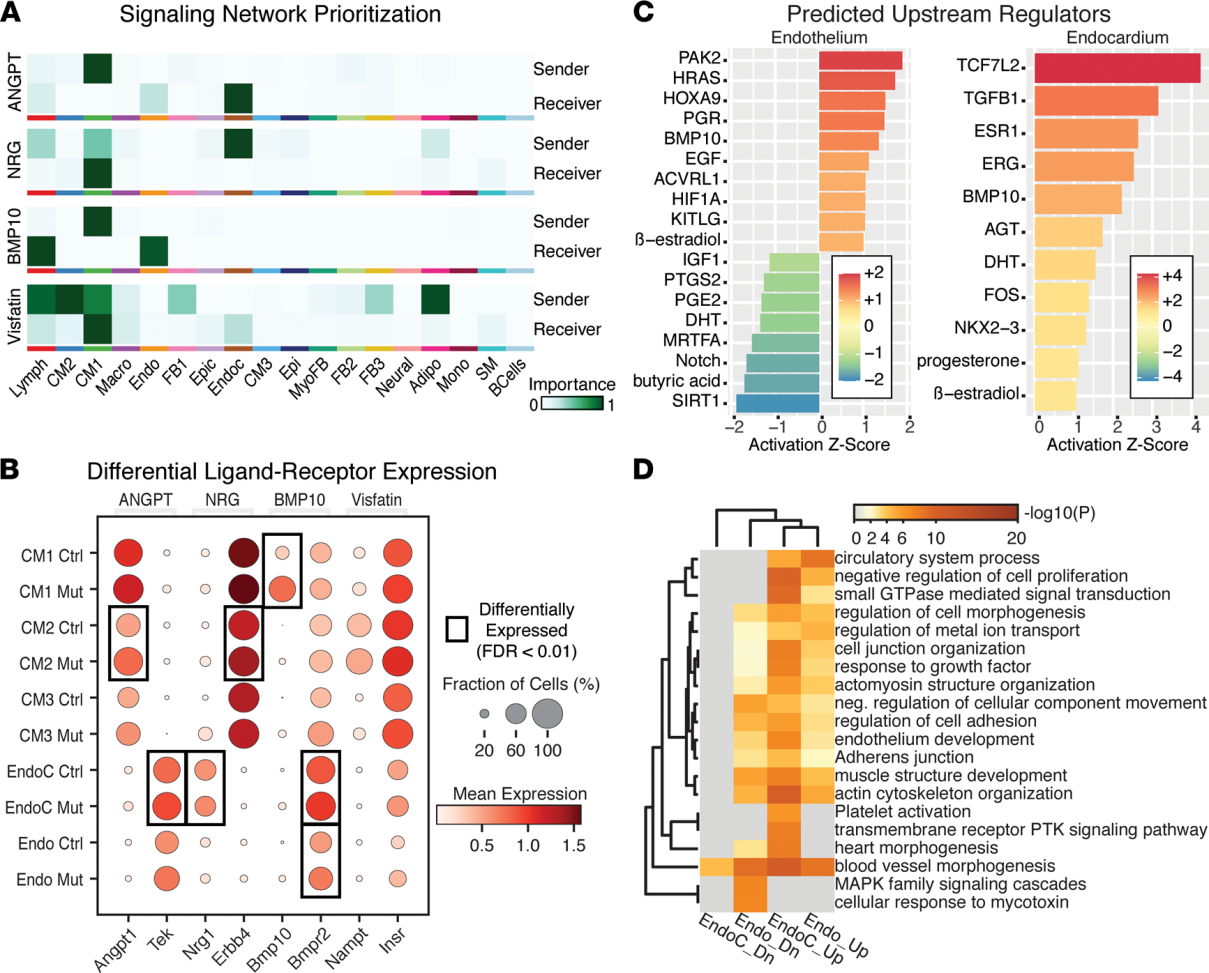
Aberrant cross-signaling between CMs and endothelium/endocardium in *Pitx2* mutant LA and PV. (**A**) Top 4 differentially expressed (control vs. *Pitx2* mutant) networks identified for CMs. (**B**) Ligand-receptor expression in CMs, endocardium (EndoC), and endothelium (Endo). Boxed pairs demonstrate significant differential expression between controls (Ctrl) and *Pitx2* mutants (Mut). (**C**) Predicted upstream regulators identified by Ingenuity Pathway Analysis (IPA) for differentially expressed genes (DEGs) comparing controls and *Pitx2* mutants for Endo (left) and EndoC (right). A positive *z* score predicts addition or activation while negative *z* score predicts subtraction or inhibition of a pathway or ligand. A complete list of DEGs can be found in Supplemental Table 8. (**D**) Top 20 GO terms for up- and downregulated DEGs for Endo and EndoC. Complete details and child terms can be found in Supplemental Table 9.

While *Pitx2* is expressed in CMs, AF also results from defects of fibroblasts, myofibroblasts, EndoC, epicardium, macrophages, and leukocytes (66–69). To investigate the cell non-autonomous effects of decreased *Pitx2* in CMs, we compared ligand-receptor interactions of CM1, CM2, and CM3 in controls and *Pitx2* mutants, irrespective of tissue source. We identified evidence for increased angiopoietin, BMP10, neuregulin, and visfatin signaling in *Pitx2* mutants (Figure 5A and Supplemental Figure 10). We found that *Pitx2*-mutant CMs had increased expression of *Angpt1* and *Bmp10*, which signal to EndoC and Endo, where expression of the respective receptors, *Tek* and *Bmpr2*, was significantly increased (Figure 5B). In the other direction, we found increased EndoC signaling of neuregulin, transcribed by *Nrg1*, to CM1 through the upregulated *Erbb4* receptor (Figure 5B).

We hypothesized that increased BMP10 or ANGPT1 outgoing signaling from CMs would change gene expression profiles of Endo and EndoC populations. We performed differential gene analysis on each of the PV and LA Endo and EndoC populations (Supplemental Table 8). First, using ingenuity pathway analysis (IPA) (70), we examined DEGs to identify potential upstream regulators of the DEGs observed (Figure 5C). Of note, this analysis identified BMP10 and ACVRL1, the type I TGF-β superfamily receptor for BMP10, in agreement with our previous analysis (Figure 5, A and B). Next, we examined functional consequences of the DEGs (38). We found widespread upregulation of a very similar complement of genes in both populations. We identified several GO pathways associated with cell-cell interactions, in particular the morphology and junctions of endothelial cells (Figure 5D and Supplemental Table 9). In other studies, these pathways have been associated with increased BMP10 signaling in both cardiac and noncardiac endothelial cells during development (71–73). Furthermore, we identified an increase in gene expression associated with platelet formation in the EndoC population of the LA (Figure 5D). As human *PITX2* variants have been associated with increases in cardioembolic stroke, this CM-to-EndoC BMP10 signaling serves as a proposed mechanism for increased AF-associated inflammation and stroke risk.

## Discussion

**Figure 6:**
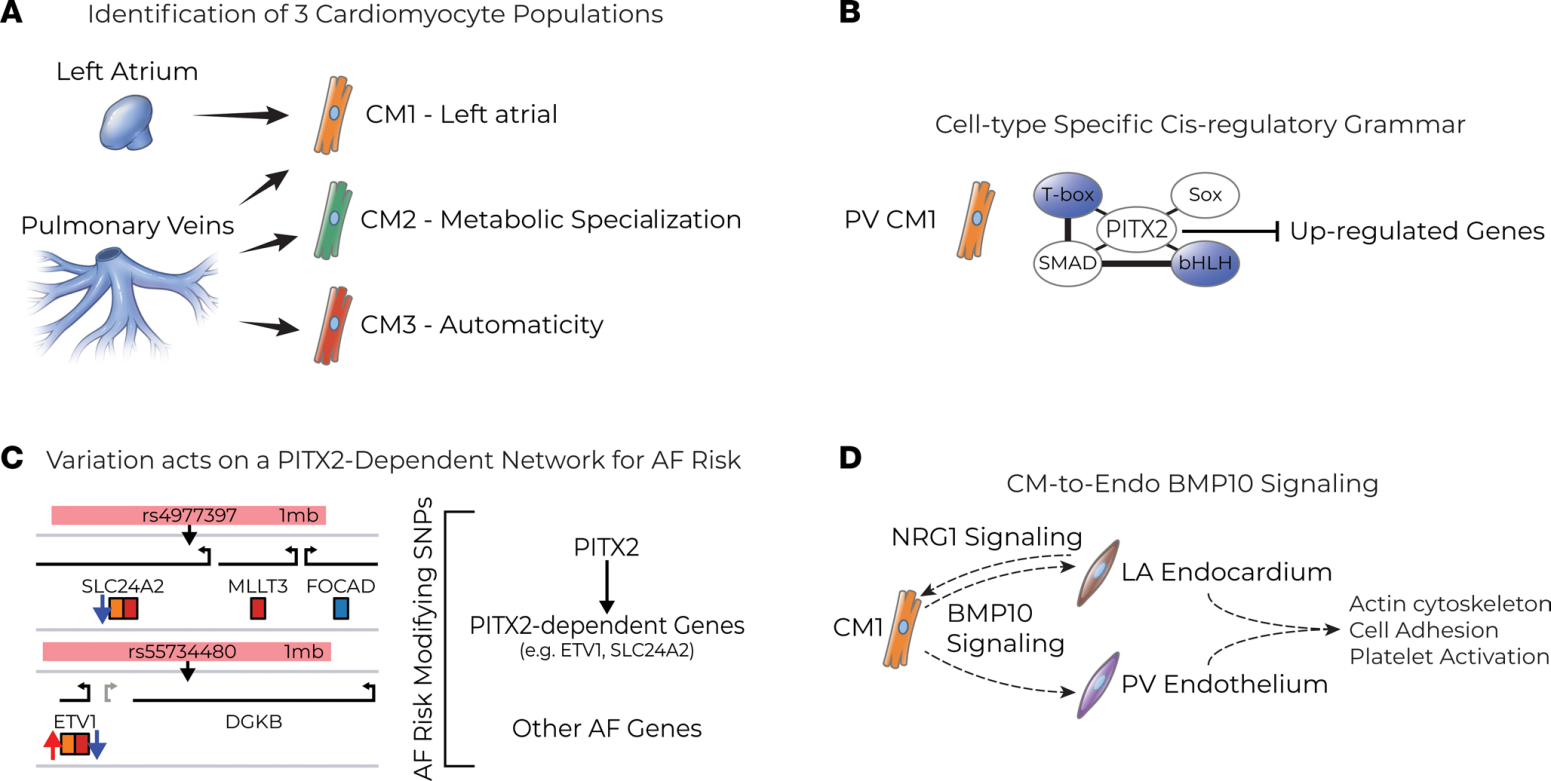
Summary of PITX2’s role in the LA and PV. (**A**) The single nuclei RNA-sequencing (snRNA-Seq) data identified 3 populations of cardiomyocytes (CMs) with 2 populations resident to the PV. The identified PV CMs make up a subset of all CMs of the PV but likely contribute to tissue-specific processes not found in the LA. (**B**) The PITX2-associated cis-regulatory grammar suggests that PITX2 interacts with a particular set of cofactors to repress gene expression in PV CM1 but interacts with those same factors in LA CM1 and PV CM2 to both activate and repress transcription. (**C**) Example loci depicting the relationship of *Pitx2*-dependent, AF-SNP–associated, cell type–enriched genes with the tagging SNP (left). Our proposed model that decreased PITX2, e.g., mouse mutants or human loss-of-function SNPs at AF-associated region (AFAR), is a potent modifier of AF risk because downstream targets of PITX2 cis-regulatory networks in each cell type are enriched in other AF risk-modifying genes (right). (**D**) LA and PV CM1 cells demonstrate substantial upregulation of *Bmp10*, which signals to the endocardium/endothelium (EndoC/Endo) of the LA/PV. EndoC/Endo of *Pitx2* mutants demonstrate differentially expressed genes (DEGs) associated with cell adhesion and platelet activation. Furthermore, the LA Endo appears to reciprocally signal to the CM1 through increased NRG1 signaling.

For over 20 years, the PV has been implicated as a common source of ectopic beats in AF (4, 74). Despite this critical role in the development of AF, the transcriptional and epigenetic regulation within the adult PV has been largely unexplored (4–6). Through this work, we examined the chromatin accessibility and transcriptional output of the mouse PVs and LA at single nuclei resolution (Figure 6). Additionally, our work explored the cell-autonomous and cell non-autonomous roles of *Pitx2*, one of the most strongly linked genes with AF in human patients (7, 8), in this context (Figure 6). This work builds upon a strong foundation of using mouse genetics to interrogate the role of *Pitx2* in AF that we have established (9, 13, 16, 17, 22), which in turn will inform future work.

In their seminal work, Haïssaguerre and colleagues demonstrated that ectopic beats originating from the PV often trigger underlying AF (4). To explain these ectopic beats, various cell types have been identified in the PVs of human patients and animal models of AF and been proposed as the source (75–78). Two of the most often proposed are a population of c-Kit^+^ interstitial Cajal cells and a population of sinoatrial node–like CMs (75–77, 79). Our analysis revealed the identity of 3 CM populations within the PVs, 2 of which are enriched in the PVs over the LA (Figure 6A). Of these 2 PV-enriched populations, CM3 is notable for having a gene expression signature consistent with electrical automaticity (Figure 6A and Supplemental Tables 2 and 3). Based on this gene expression profile, the CM3 cells appear akin to the sinoatrial node–like CMs previously reported. Furthermore, the CM3 population is enriched in several transcripts identified through AF GWAS in humans, many of which are known regulators of automaticity, e.g., *Tbx3*, or the channels that allow automaticity, e.g., *Hcn4* and *Slc24a2* (Supplemental Table 3), suggesting that this population could serve as a direct trigger source. Recent work has demonstrated regional variation of CMs of PVs underlies the ectopic foci seen in AF (80). Between this work and our own, it is apparent that there will be a keen interest in CM3 when interrogating the localization of the CM populations within the 3-dimensional space of the PV.

At the most basic level, the transcriptional perturbations we observe in *Pitx2*-mutant CMs result from the absence of PITX2 acting on CREs; however, PITX2 does not act in a vacuum, but rather within a TF milieu. Understanding the cis-regulatory landscape on which PITX2 acts will ultimately depend on the other TFs present and the regulatory elements in which PITX2 binds. The combination of TFs present in each CM type and the binding sites present at a CRE dictate the regulatory grammars acting on the target transcript (21). We utilized network-based approaches to predict the direct TF networks perturbed in *Pitx2*-mutant CMs. Our data predict that different sets of TFs working in concert generate the DEG pattern observed in the different tissues and CM subsets of *Pitx2*-mutant mice (Figure 6B). For example, our data highlight that PITX2 is likely interacting with a set of cofactors from the T-box, bHLH, Sox, and SMAD families at the upregulated genes of PV CM1 specifically (Figure 3D). This suggests PITX2 may play a stronger role in mediating transcriptional repression with this set of cofactors, such as TBX5 (16), while in the LA CM1 and PV CM2, there was no strong bias toward an activation or repression set of cofactors but instead a strong correlation in each case. Understanding the complete cis-regulatory landscape in the LA and PV CMs will serve to illuminate the basis for AF in human patients. To this point, AF-associated variants identified in GWAS are rarely associated with coding elements, but rather functional variations in CREs (51).

To leverage our AF predisposition mouse model to uncover clinically relevant biology, we compared our multiomic data with that of previously reported human AF GWAS (53). First, we identified that CMs were strongly enriched for genes associated with variation, consistent with previous reports using similar lines of inquiry in human snRNA-Seq data (29, 60). Unexpectedly, the DEGs identified in *Pitx2* mutants were commonly found among the variation-associated genes identified in the CM populations (Figure 6C). Furthermore, the DEGs associated with human variation were largely different in the different subsets (Figure 6C and Supplemental Figure 9), likely a result from the differences in PITX2 cis-regulatory grammars (Figure 3). Based on our observations, we propose a model in which decreased PITX2 acts as a potent modifier of AF risk through disrupted context-specific networks that genetically interact with other AF-associated genes (Figure 6C). Under this model, decreased *PITX2* expression, as observed in humans with loss-of-function SNPs in the AFAR locus of 4q25, results in PITX2-dependent gene expression changes, and these PITX2 targets are associated with AF GWAS hits. While some studies have found little evidence for SNP-SNP interactions in AF (81), other evidence exists that this may be the case for *PITX2* (82), and in the future a more targeted approach can be applied using the genes identified in our study.

Last, we identified that *Pitx2*-mutant CM1 cells are predicted to have increased levels of multiple outgoing signals with the strongest consistent signal for BMP10 (Figure 6D). *Bmp10* expression was strongly upregulated in the CM1 population, particularly LA CM1, where it was associated with gained DARs containing PITX2 motifs (Figure 5 and Supplemental Table 5–7). The predicted target of aberrant BMP10 signaling was the EndoC and Endo of the LA and PV, respectively, based on receptor expression and gene expression changes in those populations from *Pitx2*-mutant mice (Figure 5). *Bmp10* is expressed throughout the myocardium in development, where it is required for CM proliferation, but becomes restricted to the right atrium (RA) during late gestation (73). In development and the adult, decreased *Pitx2* is associated with increased LA *Bmp10* and has been implicated in its repression (9, 83). Furthermore, decreased levels of LA *PITX2* and increased levels of *BMP10* have been identified in human patients with AF, and increased BMP10 serum levels are correlated with relapse after cardiac ablation therapy and decreased levels with successful cardioversion (15, 84, 85). During development, increased levels of *Bmp10* expression from the heart are known to have an adverse effect on the endothelial layer of blood vessels, including premature differentiation and decreased proliferation (72, 86, 87). Furthermore, while the pulmonary circulation in the adult appears to be the primary target of BMP10 signaling from the RA (88, 89), expression of BMP10 is largely absent in the systemic circulation, suggesting a unique role in the maintenance or identity of pulmonary vasculature that may interfere with the identity or maintenance of the vasculature in the brain or other parts of the body that may make them prone to thrombus formation or damage (90). Indeed, GO term analysis showed increased gene expression consistent with clot formation in the LA EndoC (Figure 5D). The 4q25 locus is strongly associated with both AF and cardioembolic stroke risk. The stroke risk is attributed to clot formation in the fibrillating atrial chambers (91–93). Additionally, BMP10 has been demonstrated to drive *NRG1* expression in the EndoC specifically (71), while NRG1 is known to regulate and drive *Etv1* expression in LA CMs (94, 95). Our cell-cell communication analysis demonstrated both an increase in CM1-to-EndoC BMP10 signaling and EndoC-to-CM1 NRG1 signaling (Figure 5B), suggesting *Pitx2*-dependent signaling feedback that may be driving the increased *Etv1* expression in CM1 (Figure 6D). This increased atrial *Etv1* may contribute to AF predisposition in the CMs (96). Our work suggests that this could be multifactorial susceptibility in which decreased *PITX2* and increased BMP10 in the LA generates prothrombotic conditions and stroke risk predisposition.

We believe our data suggest developmental PITX2 acts to pattern the cis-regulatory grammars of the LA and PV CMs. In situations with decreased PITX2, i.e., mouse mutants or humans with loss-of-function SNPs in AFAR, these cis-regulatory grammars are perturbed. In combination with external stressors, e.g., age, and other genetic predispositions, these perturbed cis-regulatory grammars create an increased lifetime risk for AF development.

## Methods

### Mouse lines.

The *Tg(Ckmm-cre)5Khn*/0 (*MCK-cre*) mice were crossed to the germline *Pitx2^tm1Jfm^* (*Pitx2^–^*) allele to generate *MCK-cre* *Pitx2*^+/–^ mice, which were crossed with homozygous *Pitx2^tm1.1Sac^* (*Pitx2*^fl/fl^) to generate the *Pitx2* control (*Pitx2*^fl/+^) and *Pitx2*-mutant (*MCK-cre* *Pitx2*^fl/–^) mice (97–99). Mice were raised to adulthood (6–8 months) and genotypes confirmed by PCR prior to experimentation. 

### snRNA-Seq and snATAC-Seq.

For snRNA-Seq and snATAC-Seq, the LA and the PVs were dissected from the adult mouse heart. The left atrial dissection included both the chamber and the appendage, while the PVs included the proximal connection to the atrium, the primary PVs, and the branches prior to entry into the lungs.

Nuclei were harvested from the 2 tissue sources in pools of 8 *Pitx2* control and 8 *Pitx2*-mutant mice using a previously published method with modifications (100). In brief, the tissues were minced with sharp scissors in 250 μL of HB++ (HB: 0.25 M sucrose, 25 mM KCl, 5 mM MgCl_2_, 20 mM Tricine-KOH, pH 7.8; ++: 0.15 mM spermine, 0.5 mM spermidine, 1 mM DTT) and centrifuged at 500*g* for 5 minutes at room temperature to remove excess red blood cells and fat and replaced with 250 μL of fresh HB++. Sample was transferred to a 2 mL Dounce homogenizer and the tube washed with an additional 250 μL of HB++ to collect any remaining sample and transferred. Tissue was homogenized 30 times with loose and tight pestle before adding 32 μL of 5% NP-40 (in HB) and dounced with tight pestle an additional 75 times. Sample was strained through a 40 μM strainer and washed with 9.5 mL of NWB (PBS with 1% BSA and 0.2 U RNase inhibitor from Promega) and spun down at 500*g* at 4°C for 5 minutes. The supernatant was removed and nuclei were resuspended in 500 μL of NWB and 900 μL of SCB (90% Nuclei PURE 2M Sucrose Cushion Solution and 10% Nuclei PURE Sucrose Cushion Solution; MilliporeSigma NUC-201). Then 1400 μL of nuclei suspension was layered on top of 500 μL SCB in a 2 mL Eppendorf LoBind tube and centrifuged at 13,000*g* at 4°C for 45 minutes. The supernatant was removed, leaving approximately 50 μL of nuclei pellet, which was resuspended in 1 mL of NWB. Nuclei were quantified using the INCYTO C-Chip DHC-F01.

The snRNA-Seq libraries were generated using the 10x Genomics Chromium Single Cell 3’ v3 reagent kit, and in parallel the snATAC-Seq libraries were generated using the Chromium Single Cell ATAC v1 with a target of 10,000 nuclei per library according to the manufacturer’s instructions. The libraries were sequenced on an Illumina NovaSeq6000 with the Genome and RNA Profiling Core at Baylor College of Medicine and an Illumina NextSeq 500. Raw sequencing data were handled by the 10x Genomics Cell Ranger software (cellranger-3.0.1 and cellranger-atac-1.2.0) and mapped to the mouse mm10 genome. For snRNA-Seq, the gene counts were quantified using Cell Ranger count to the mouse transcriptome including pre-mRNA (v3.0.0) and passed to Seurat (25–27) for downstream analysis. For snATAC-Seq, samples were individually aligned using cellranger-atac count and aggregated using cellranger-atac aggr (--nosecondary --normalize=none) using the peak output from each individual sample. Following initial clustering (see below for downstream analysis) and per-cluster and per-sample peak calling using MACS2 callpeak (v2.1.1.20160309) with parameters -f BAMPE -g mm -B -q 0.1 (101, 102), samples were reaggregated using cellranger-atac aggr using a union set of peaks and passed to Seurat and Signac (26, 28) for downstream analysis. Raw sequencing data and other associated files have been uploaded to the National Center for Biotechnology Information’s (NCBI) Gene Expression Omnibus (GSE183310).

### snRNA-Seq analysis cell quality control and filtering.

After performing snRNA-Seq, preprocessing steps were performed using CellRanger 3.0.1 followed by postprocessing using CellBender v0.1 (24) with the remove-background module in default settings. This allowed the removal of ambient RNA background molecules and random barcode swapping from (raw) snRNA-Seq gene-by-cell count matrices. Each data source was processed and annotated separately to account for source-specific quality differences. These metrics are included as covariates for further processing. Single nuclei were filtered (Supplemental Figure 1) for unique molecular identifier (UMI) counts (500 < UMI counts < 25,000), genes (no genes > 500), mitochondrial genes (mitochondrial % < 5%), and doublet score (doublet score < 0.25). Seurat toolkit version v.4.0 (26) in R v.3.8 was used to perform downstream analyses. The UMI count matrices of cells that passed previous filtering were normalized using the Seurat function SCTransform regressing out unwanted sources of variation (mitochondrial % and cell cycle scoring). For each sequenced library we calculated the top 5000 most variable features using FindVariableFeatures.

### Batch alignment.

We found anchors across subsets, also known as mutual nearest neighbors, using the Seurat function FindIntegrationAnchors. We then integrated using canonical correlation analysis (CCA) as implemented in Seurat using the IntegrateData function (26). In brief, the algorithm first jointly reduces the dimensionality of both data sets using diagonalized CCA, then applies L2 normalization to the canonical correlation vectors, and finally searches for mutual nearest neighbors in this shared low-dimension representation. Ultimately, an integrated matrix is constructed for each cell applying a correction vector (based on anchor score and similarity score) (26).

### Principal component analysis and unsupervised clustering.

The resulting integrated data were scaled, and principal component analysis (PCA) was performed to reduce the dimension into 100 principal components (PCs). Clusters were identified using k-nearest-neighbor-graph based clustering implemented in Seurat as FindNeighbors and FindClusters. We performed a grid search of different combinations of parameters, aiming to optimize the biological relevance of the resulting clusters. Results yielded a clustering based on the top 50 PCs, and using a resolution of 0.8 maximized both our estimation of biological relevance and numbers of clusters obtained.

### DEGs.

To annotate the populations and subpopulations of each cell cluster and comparison between genotypes, we calculated the DEGs using the Wilcoxon rank sum test as implemented in the Seurat workflow as FindMarkers. A gene is differentially expressed if it has a log_2_ fold change > 0.25 and an FDR < 0.01, unless stated otherwise.

### Cell-cell interactions.

A systematic analysis of cell communication was based on the network analysis and pattern recognition approaches provided by CellChat v.1.0.0 R package (103). We used the standard workflow to predict major signaling inputs and outputs of cells and how these cells and signals coordinate for functions. Subsequently, we classified signaling pathways and depicted conserved and context-specific pathways between controls and *Pitx2* mutants.

### GO term and pathway analysis.

All GO term analyses were performed using Metascape (38) using the multiple gene list comparison function with default settings. Predicted upstream pathway analysis was performed using IPA (Qiagen) for control versus *Pitx2*-mutant DEGs identified from each of the Endo and EndoC populations (70).

### snATAC-Seq quality control.

We removed low-quality snATAC-Seq cells based on quality control metrics (Supplemental Figure 1) (26, 28). We first filtered out the cells that had fewer than 15% of fragments (reads) in peaks. These cells with low total fragments falling within ATAC-Seq peaks may represent technical artifacts or low-quality cells that need to be removed. We also removed cells with peak region fragments greater than 30,000 and less than 1500. Cells with few reads have low sequencing depth, while cells with extremely high reads often represent nuclear clumps or doublets (26).

### Harmony alignment and unsupervised clustering.

To align the 4 snATAC-Seq data sets, we utilized the RunHarmony function using lsi reduction on all peaks (26, 28). Similar to the snRNA-Seq, the resulting aligned and integrated data were reduced to 50 PCs. Clusters were identified using k-nearest-neighbor-graph–based clustering implemented in Seurat as FindNeighbors and FindClusters using the Harmony reduction (26, 28). We performed a grid search of different combinations of parameters, aiming to optimize the biological relevance of the resulting clusters. Results yielded a clustering based on the top 50 PCs, and using a resolution of 1.25 maximized both our estimation of biological relevance and numbers of clusters obtained. Following this, a UMAP was generated using the RunUMAP function with the Harmony reduction graph and a repulsion strength of 2 (26, 28).

### Label transfer.

To transfer labels from snRNA-Seq to snATAC-Seq (Supplemental Figure 3), we first identified the common correlation patterns in the gene activity matrix derived from the snATAC-Seq data and snRNA-Seq data. This gene activity matrix was created by summing the fragments that intersected with the gene body and promoter region. This calculation was performed using the GeneActivity function in the Seurat (26, 28), which enables mapping of fragments of each cell to the 2 kb region upstream of gene coordinates. In the integration step, we identified patterns in the biological states of 2 modalities using FindTransferAnchors function. Here, we used the Harmony algorithm, which iteratively corrects PCA embeddings, for dimensionality reduction when finding anchors. Then, we used the reference cell type labels defined in snRNA-Seq data to assign scores to each snATAC-Seq nuclei. For each cluster identified in the UMAP, we assigned the plurality cell type for those cells assigned to different cell type yet in the same cluster (Supplemental Figure 3).

### Computing NMSs.

We first identified the peaks that had the PITX2 motif occurrences using MEME suite tool FIMO with *P* value cutoff of 1 × 10^–4^ and CisBP (v2.0.0) database (104, 105). For every DEG and every PITX2-containing peak, we assigned it to a TAD/inter-TAD space using published data (40, 41). We then assigned the PITX2-containing peaks to DEGs within the same TAD/inter-TAD space as putative CREs for that target gene. We also calculated the accessibility count for an “average” single nuclei in LA and PV using the Seurat:AverageExpression(slot=“counts”) function. Briefly, the method works as follows: first, for every peak of PITX2 occurrence, we multiply the accessibility score A_i_ with the number of PITX2 occurrences O_i_ and weighted by the peak length L_i_. The NMS was defined as: NMS_i_ = ([A_i_ × O_i_]/L_i_) × 10,000. For purposes of identifying only accessible regions in the LA or PV CM1 or CM2 populations, we defined active sites as those with NMS > 1.

### DAR motif analysis.

To identify motifs associated with CM1 and CM2 DARs, we utilized the findMotifsGenome function from HOMER (106). To do this, we used the CM2 DARs as the background for the CM1 analysis and CM1 DARs as the background for CM2 in order to find the motifs that make the biggest difference. The list of known motifs was ranked by *q* value, and the top 3 unique motifs, i.e., those representing unique families of TFs, were selected for further examination. All motifs shown have a *q* value < 3 × 10^–4^.

### PITX2 TF-TF colocalization and network analysis.

To understand the transcriptional cis-regulatory grammar in the context of PITX2 motifs, we performed a series of analyses on the peaks with PITX2 motif hits. We retained the motif families present in the CisBP (v2.0.0) database that hit those peaks identified by FIMO with a *P* value cutoff 1 × 10^–4^ (104, 105). TF motif families were defined using published annotations (105, 107). We calculated the Pearson’s correlation coefficient for each motif family (Supplemental Figure 6). We created a network representation of the motifs that co-occurred in PITX2-associated peaks (Supplemental Figure 7). Each node represents a motif family, and the edges represent the colocalization across peaks. To find communities in the graph, we performed spectral community detection (108). This method iteratively calculates the eigenvector of the modularity matrix for the largest positive eigenvalue and then separates vertices into communities based on the corresponding element in the eigenvector (108). R v.3.8 and Igraph v1.2.6 were used to perform these analyses.

### Footprinting analysis.

In order to generate a list of DNA footprinted sites using the snATAC-Seq, we utilized the TOBIAS package (45). To do this, we extracted the reads corresponding to each of the LA WT CM1, PV WT CM1, and PV WT CM2 to generate pseudo-bulk ATAC-Seq library files. The TOBIAS ATACorrect function was run on each of these pseudo-bulk bam files against the reference genome at the list of all CM peaks and ENCODE blacklist sites (109). Following this step, the TOBIAS FootprintScores function was run for LA WT CM1, PV WT CM1, and PV WT CM2 corrected signals in the list of all CM peaks. Finally, the TOBIAS BINDetect function was run using the CisBP database (105) in order to generate a list of all sites containing a PITX2 footprint.

### Examination of public databases.

The predicted and validated CREs were pulled from VISTA (46) and ENCODE (47). For VISTA, validated cardiac CREs were defined as “VISTA Cardiac Enhancer” while noncardiac validated CREs were defined as “VISTA Other Enhancer” (Supplemental Figure 5B). For the ENCODE list of candidate CREs, the complete list was pulled from ENCFF904ZZH, while the P0 and 8-week-old whole heart sets are from ENCFF509ZNV and ENCFF656HRU, respectively (47). The list of sites used in overlap fit the criteria of promoter-like signature; TSS-proximal, enhancer-like signature; or TSS-distal, enhancer-like signature (Supplemental Figure 5B).

### Comparison of human GWAS with mouse snRNA-Seq.

Human AF SNPs (EFO_0000275) were retrieved from the GWAS catalog on January 23, 2021 (53). Association of AF SNPs with genes was performed using MAGMA and the provided NCBI38 annotation to associate TSSs within ± 500 kbp of the tagging SNP (54). Mouse-to-human gene transfer was performed using the union of the Ensembl database (release 103) and NCBI HomoloGene catalog (build 68) (55–58). Identification of cluster enriched gene expression and within cell cluster expression binning was performed as previously described using 25 equally sized bins and a 0 bin for genes with no expression (52). Enriched cluster expression was identified by 1-sided Welch’s 2-sample *t* test followed by multiple testing correction as many cell types demonstrated unequal variance between SNP-associated and non-SNP-associated gene bins.

### Statistics.

For comparing cluster proportion differences, we used a 2-tailed χ^2^ test followed by multiple-testing correction; a comparison was considered significant at FDR < 1 × 10^–5^ (Figure 1, D and E). For snRNA-Seq analyses, DEGs were identified using the Wilcoxon rank sum test as implemented in the Seurat workflow as FindMarkers (Supplemental Tables 1, 2, 5, and 8). A gene is differentially expressed if it has a log_2_ fold change > 0.25 and an FDR < 0.01, unless stated otherwise. Tests of enrichment (Figure 2H and Figure 4E) utilized 2-tailed FET followed by multiple-testing correction. An FDR < 0.01 was considered significant. Identification of footprint enrichment was done using an unpaired, 2-tailed Wilcoxon rank sum test (Supplemental Figure 5A). Test of PITX2 NMS enrichment at validated and predicted CREs utilized the 2-sided FET followed by multiple-testing correction (Supplemental Figure 5B). An FDR < 0.01 was considered significant while an FDR < 1 × 10^–10^ was considered very significant. Identification of motif-motif correlation (Supplemental Figure 6) utilized the Pearson’s correlation coefficient. Enriched cluster expression (Figure 4A) was identified by 1-sided Welch’s 2-sample *t* test followed by multiple-testing correction.

### Study approval.

All murine experiments were approved and performed under the Baylor College of Medicine Institutional Animal Care and Use Committee protocol number 5713.

## Author contributions

JDS and JFM conceived the project. JDS, FJGC, MP, MAHS, and JFM conceived and implemented the computational methods. JDS and ZAK performed the experiments. MAHS and JFM supervised the project. JDS and JFM prepared the manuscript with critical review and input from all authors.

## Supplementary Material

Supplemental data

Supplemental table 1

Supplemental table 2

Supplemental table 3

Supplemental table 4

Supplemental table 5

Supplemental table 6

Supplemental table 7

Supplemental table 8

Supplemental table 9

## Version 1 04/26/2022

In-Press Preview

## Version 2 06/08/2022

Electronic publication

## References

[1] Staerk L (2018). Lifetime risk of atrial fibrillation according to optimal, borderline, or elevated levels of risk factors: cohort study based on longitudinal data from the Framingham Heart Study. BMJ.

[2] Andrade J (2014). The clinical profile and pathophysiology of atrial fibrillation: relationships among clinical features, epidemiology, and mechanisms. Circ Res.

[3] Diener H-C (2019). Atrial fibrillation and cognitive function: JACC review topic of the week. J Am Coll Cardiol.

[4] Haïssaguerre M (1998). Spontaneous initiation of atrial fibrillation by ectopic beats originating in the pulmonary veins. N Engl J Med.

[5] Boutilier JK (2017). Gene expression networks in the murine pulmonary myocardium provide insight into the pathobiology of atrial fibrillation. G3 (Bethesda).

[7] Roselli C (2018). Multi-ethnic genome-wide association study for atrial fibrillation. Nat Genet.

[8] Malik R (2018). Multiancestry genome-wide association study of 520,000 subjects identifies 32 loci associated with stroke and stroke subtypes. Nat Genet.

[9] Zhang M (2019). Long-range Pitx2c enhancer-promoter interactions prevent predisposition to atrial fibrillation. Proc Natl Acad Sci U S A.

[10] Liu C (2001). Regulation of left-right asymmetry by thresholds of Pitx2c activity. Development.

[11] Liu C (2002). Pitx2c patterns anterior myocardium and aortic arch vessels and is required for local cell movement into atrioventricular cushions. Development.

[12] Ai D (2006). Pitx2 regulates cardiac left-right asymmetry by patterning second cardiac lineage-derived myocardium. Dev Biol.

[13] Wang J (2010). Pitx2 prevents susceptibility to atrial arrhythmias by inhibiting left-sided pacemaker specification. Proc Natl Acad Sci U S A.

[14] Kirchhof P (2011). PITX2c is expressed in the adult left atrium, and reducing Pitx2c expression promotes atrial fibrillation inducibility and complex changes in gene expression. Circ Cardiovasc Genet.

[15] Reyat JS (2020). Reduced left atrial cardiomyocyte PITX2 and elevated circulating BMP10 predict atrial fibrillation after ablation. JCI Insight.

[16] Nadadur RD (2016). Pitx2 modulates a Tbx5-dependent gene regulatory network to maintain atrial rhythm. Sci Transl Med.

[17] Van Ouwerkerk AF (2020). Epigenetic and transcriptional networks underlying atrial fibrillation. Circ Res.

[18] Bürglin TR, Affolter M (2016). Homeodomain proteins: an update. Chromosoma.

[19] Tao G (2016). Pitx2 promotes heart repair by activating the antioxidant response after cardiac injury. Nature.

[20] Junion G (2012). A transcription factor collective defines cardiac cell fate and reflects lineage history. Cell.

[21] Avsec Ž (2021). Base-resolution models of transcription-factor binding reveal soft motif syntax. Nat Genet.

[22] Tao Y (2014). Pitx2, an atrial fibrillation predisposition gene, directly regulates ion transport and intercalated disc genes. Circ Cardiovasc Genet.

[23] Lamas GA (2002). Ventricular pacing or dual-chamber pacing for sinus-node dysfunction. N Engl J Med.

[25] Butler A (2018). Integrating single-cell transcriptomic data across different conditions, technologies, and species. Nat Biotechnol.

[26] Stuart T (2019). Comprehensive integration of single-cell data. Cell.

[27] Hafemeister C, Satija R (2019). Normalization and variance stabilization of single-cell RNA-seq data using regularized negative binomial regression. Genome Biol.

[29] Tucker NR (2020). Transcriptional and cellular diversity of the human heart. Circulation.

[30] Yamada N (2019). Mutant KCNJ3 and KCNJ5 potassium channels as novel molecular targets in bradyarrhythmias and atrial fibrillation. Circulation.

[31] Hodgson-Zingman DM (2008). Atrial natriuretic peptide frameshift mutation in familial atrial fibrillation. N Engl J Med.

[32] Barana A (2014). Chronic atrial fibrillation increases microRNA-21 in human atrial myocytes decreasing L-type calcium current. Circ Arrhythm Electrophysiol.

[33] Stevens J (2010). Analysis of the asymmetrically expressed Ablim1 locus reveals existence of a lateral plate Nodal-independent left sided signal and an early, left-right independent role for nodal flow. BMC Dev Biol.

[34] Hotter G (2020). The influenza virus NS1A binding protein gene modulates macrophages response to cytokines and phagocytic potential in inflammation. Sci Rep.

[35] Hojayev B (2012). FHL2 binds calcineurin and represses pathological cardiac growth. Mol Cell Biol.

[36] Gergely F (2000). The TACC domain identifies a family of centrosomal proteins that can interact with microtubules. Proc Natl Acad Sci U S A.

[37] Chandler NJ (2009). Molecular architecture of the human sinus node: insights into the function of the cardiac pacemaker. Circulation.

[38] Zhou Y (2019). Metascape provides a biologist-oriented resource for the analysis of systems-level datasets. Nat Commun.

[39] Mueller-Hoecker J (2008). Of rodents and humans: a light microscopic and ultrastructural study on cardiomyocytes in pulmonary veins. Int J Med Sci.

[40] Wang Q (2018). Sub-kb Hi-C in D. melanogaster reveals conserved characteristics of TADs between insect and mammalian cells. Nat Commun.

[41] Bonev B (2017). Multiscale 3D genome rewiring during mouse neural development. Cell.

[42] Steimle JD, Moskowitz IP (2017). TBX5: a key regulator of heart development. Curr Top Dev Biol.

[43] Huppke P (2017). Activating de novo mutations in NFE2L2 encoding NRF2 cause a multisystem disorder. Nat Commun.

[44] Yang Y (2021). A missense mutation in Pitx2 leads to early-onset glaucoma via NRF2-YAP1 axis. Cell Death Dis.

[45] Bentsen M (2020). ATAC-seq footprinting unravels kinetics of transcription factor binding during zygotic genome activation. Nat Commun.

[46] Visel A (2006). VISTA Enhancer Browser—a database of tissue-specific human enhancers. Nucleic Acids Res.

[47] ENCODE Project Consortium (2020). Expanded encyclopaedias of DNA elements in the human and mouse genomes. Nature.

[48] Suszko MI (2008). Smad3 and Pitx2 cooperate in stimulation of FSHbeta gene transcription. Mol Cell Endocrinol.

[49] Berry FB (2006). Functional interactions between FOXC1 and PITX2 underlie the sensitivity to FOXC1 gene dose in Axenfeld-Rieger syndrome and anterior segment dysgenesis. Hum Mol Genet.

[50] Cao H (2010). Tbx1 regulates progenitor cell proliferation in the dental epithelium by modulating Pitx2 activation of p21. Dev Biol.

[51] Van Ouwerkerk AF (2020). Identification of functional variant enhancers associated with atrial fibrillation. Circ Res.

[52] Skene NG (2018). Genetic identification of brain cell types underlying schizophrenia. Nat Genet.

[53] Buniello A (2019). The NHGRI-EBI GWAS catalog of published genome-wide association studies, targeted arrays and summary statistics 2019. Nucleic Acids Res.

[54] De Leeuw CA (2015). MAGMA: generalized gene-set analysis of GWAS data. PLoS Comput Biol.

[55] Durinck S (2005). BioMart and Bioconductor: a powerful link between biological databases and microarray data analysis. Bioinformatics.

[56] Durinck S (2009). Mapping identifiers for the integration of genomic datasets with the R/Bioconductor package biomaRt. Nat Protoc.

[57] NCBI Resource Coordinators (2016). Database resources of the National Center for Biotechnology Information. Nucleic Acids Res.

[58] Howe KL (2021). Ensembl 2021. Nucleic Acids Res.

[59] Kolishovski G (2019). The JAX Synteny Browser for mouse-human comparative genomics. Mamm Genome.

[60] Litviňuková M (2020). Cells of the adult human heart. Nature.

[61] Scott L (2021). NLRP3 inflammasome is a key driver of obesity-induced atrial arrhythmias. Cardiovasc Res.

[63] Gudbjartsson DF (2007). Variants conferring risk of atrial fibrillation on chromosome 4q25. Nature.

[64] Lubitz SA (2010). Independent susceptibility markers for atrial fibrillation on chromosome 4q25. Circulation.

[65] Shoemaker MB (2015). Common genetic variants and response to atrial fibrillation ablation. Circ Arrhythm Electrophysiol.

[66] Hohmann C (2020). Inflammatory cell infiltration in left atrial appendageal tissues of patients with atrial fibrillation and sinus rhythm. Sci Rep.

[67] Nattel S (2017). Molecular and cellular mechanisms of atrial fibrosis in atrial fibrillation. JACC Clin Electrophysiol.

[68] De Groot N (2016). Direct proof of endo-epicardial asynchrony of the atrial wall during atrial fibrillation in humans. Circ Arrhythm Electrophysiol.

[69] Shahid F (2018). Role of monocytes in heart failure and atrial fibrillation. J Am Heart Assoc.

[70] Krämer A (2014). Causal analysis approaches in Ingenuity Pathway Analysis. Bioinformatics.

[71] Mikryukov AA (2021). BMP10 signaling promotes the development of endocardial cells from human pluripotent stem cell-derived cardiovascular progenitors. Cell Stem Cell.

[72] Laux DW (2013). Circulating Bmp10 acts through endothelial Alk1 to mediate flow-dependent arterial quiescence. Development.

[73] Chen H (2004). BMP10 is essential for maintaining cardiac growth during murine cardiogenesis. Development.

[74] Jaïs P (1997). A focal source of atrial fibrillation treated by discrete radiofrequency ablation. Circulation.

[75] Ye W (2015). Genetic regulation of sinoatrial node development and pacemaker program in the venous pole. J Cardiovasc Dev Dis.

[76] Morel E (2008). Identification and distribution of interstitial Cajal cells in human pulmonary veins. Heart Rhythm.

[77] He X-Z (2012). Cardiomyocyte progenitors in a canine pulmonary vein model of persistent atrial fibrillation. J Cardiol.

[78] Jones SA (2008). Distinguishing properties of cells from the myocardial sleeves of the pulmonary veins: a comparison of normal and abnormal pacemakers. Circ Arrhythm Electrophysiol.

[79] Gherghiceanu M (2008). Interstitial Cajal-like cells (ICLC) in myocardial sleeves of human pulmonary veins. J Cell Mol Med.

[80] Potekhina VM (2019). The local repolarization heterogeneity in the murine pulmonary veins myocardium contributes to the spatial distribution of the adrenergically induced ectopic foci. J Physiol Sci.

[81] Lin H (2016). Gene-gene interaction analyses for atrial fibrillation. Sci Rep.

[82] Huang Y (2015). Molecular basis of gene-gene interaction: cyclic cross-regulation of gene expression and post-GWAS gene-gene interaction involved in atrial fibrillation. PLoS Genet.

[83] Tessari A (2008). Myocardial Pitx2 differentially regulates the left atrial identity and ventricular asymmetric remodeling programs. Circ Res.

[84] Chua W (2021). Dynamic changes of cardiovascular biomarkers after ablation for atrial fibrillation: observations from AXAFA-AFNET5. Eur Heart J.

[85] Meyre PB (2022). Biomarkers associated with rhythm status after cardioversion in patients with atrial fibrillation. Sci Rep.

[86] Capasso TL (2020). BMP10-mediated ALK1 signaling is continuously required for vascular development and maintenance. Angiogenesis.

[87] Levet S (2015). BMP9 and BMP10 are necessary for proper closure of the ductus arteriosus. Proc Natl Acad Sci U S A.

[88] Morrell NW (2019). Finding the needle in the haystack: BMP9 and 10 emerge from the genome in pulmonary arterial hypertension. Eur Respir J.

[89] Bouvard C Different cardiovascular and pulmonary phenotypes for single- and double-knock-out mice deficient in BMP9 and BMP10. Cardiovasc Res.

[90] Mitrofan C-G (2017). Bone morphogenetic protein 9 (BMP9) and BMP10 enhance tumor necrosis factor-α-induced monocyte recruitment to the vascular endothelium mainly via activin receptor-like kinase 2. J Biol Chem.

[91] Pulit SL (2018). Atrial fibrillation genetic risk differentiates cardioembolic stroke from other stroke subtypes. Neurol Genet.

[92] Gretarsdottir S (2008). Risk variants for atrial fibrillation on chromosome 4q25 associate with ischemic stroke. Ann Neurol.

[93] Lemmens R (2010). The association of the 4q25 susceptibility variant for atrial fibrillation with stroke is limited to stroke of cardioembolic etiology. Stroke.

[94] Shekhar A (2018). ETV1 activates a rapid conduction transcriptional program in rodent and human cardiomyocytes. Sci Rep.

[95] Yamaguchi N (2021). Cardiac pressure overload decreases ETV1 expression in the left atrium, contributing to atrial electrical and structural remodeling. Circulation.

[96] Rommel C (2018). The transcription factor ETV1 induces atrial remodeling and arrhythmia. Circ Res.

[97] Lu M-F (1999). Function of Rieger syndrome gene in left-right asymmetry and craniofacial development. Nature.

[98] Gage PJ (1999). Dosage requirement of Pitx2 for development of multiple organs. Development.

[99] Brüning JC (1998). A muscle-specific insulin receptor knockout exhibits features of the metabolic syndrome of NIDDM without altering glucose tolerance. Mol Cell.

[100] Mo A (2015). Epigenomic signatures of neuronal diversity in the mammalian brain. Neuron.

[101] Zhang Y (2008). Model-based analysis of ChIP-seq (MACS). Genome Biol.

[102] Feng J (2012). Identifying ChIP-seq enrichment using MACS. Nat Protoc.

[103] Jin S (2021). Inference and analysis of cell-cell communication using CellChat. Nat Commun.

[104] Grant CE (2011). FIMO: scanning for occurrences of a given motif. Bioinformatics.

[105] Weirauch MT (2014). Determination and inference of eukaryotic transcription factor sequence specificity. Cell.

[106] Heinz S (2010). Simple combinations of lineage-determining transcription factors prime cis-regulatory elements required for macrophage and B cell identities. Mol Cell.

[107] Wingender E (2013). TFClass: an expandable hierarchical classification of human transcription factors. Nucleic Acids Res.

[108] Newman MEJ (2013). Spectral methods for community detection and graph partitioning. Phys Rev E Stat Nonlin Soft Matter Phys.

[109] Amemiya HM (2019). The ENCODE blacklist: identification of problematic regions of the genome. Sci Rep.

