# Supplementary material for: Decoding the PITX2-controlled genetic network in atrial fibrillation
Source: JCI Insight. 2022 Jun 8;7(11):e158895. doi: 10.1172/jci.insight.158895 (PMC9221021; doi:10.1172/jci.insight.158895)
Supplement: Supplemental data [file jciinsight-7-158895-s054.pdf]

SUPPLEMENTARY FIGURES

Figure S1

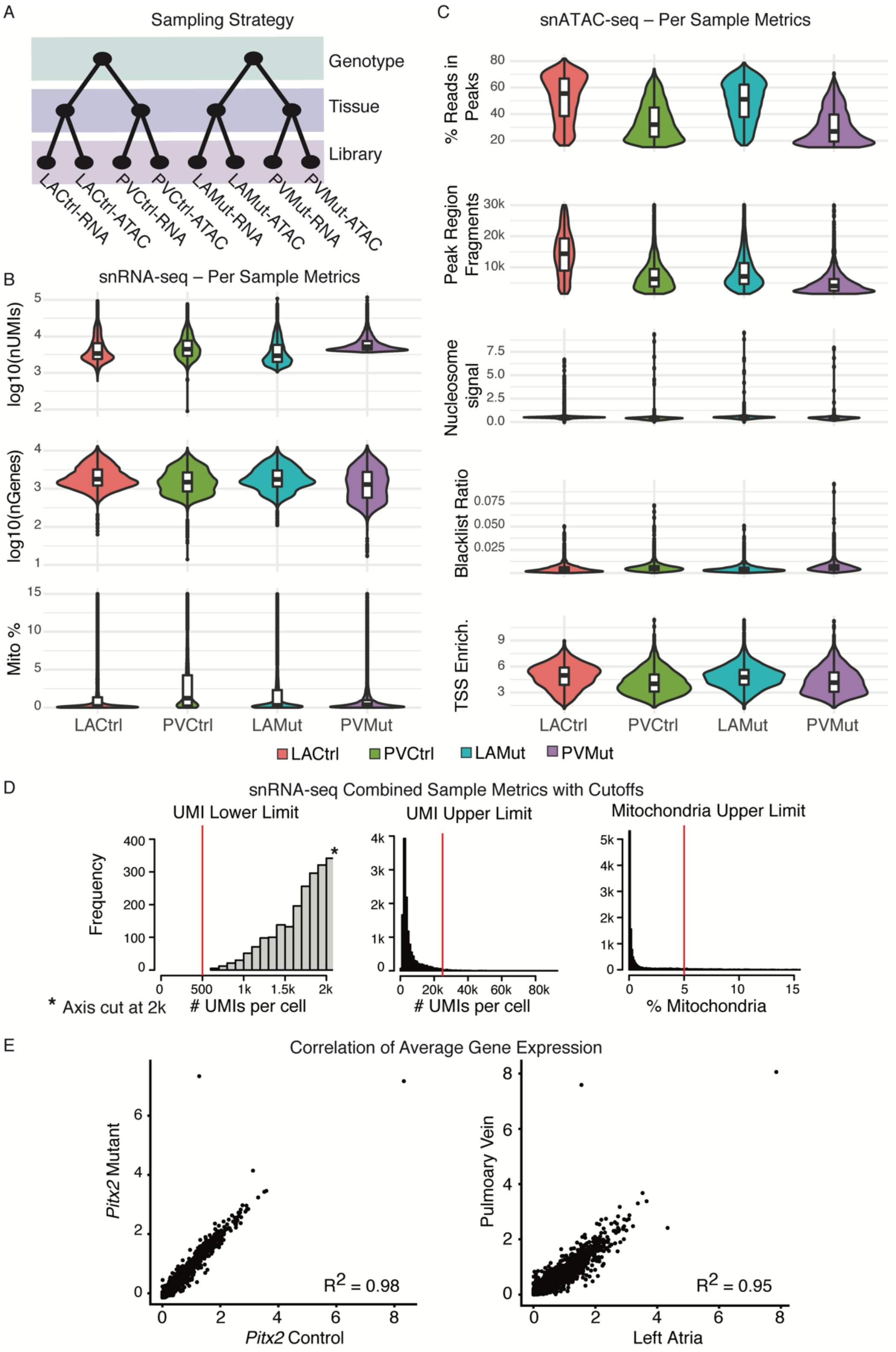

Figure S1. Quality control and data cut-offs for single nuclei profiling

A. Graphic depicting the origin of each of the 8 single-nuclei (sn) datasets. Left atrial (LA) or pulmonary vein (PV) tissue was harvested from 8 control and 8 *Pitx2* mutant mice. After nuclei were isolated, a subset of nuclei was utilized for snRNA-seq and snATAC-seq library preparations. B. Sample metrics for the snRNA-seq libraries examining number of UMIs per nuclei (nUMIs), number of genes per nuclei (nGenes), and percentage of reads from mitochondria (Mito %). C. Sample metrics for the snATAC-seq libraries examining percent of reads in peaks, number of fragments in peaks, percent of reads in nucleosome, ratio of reads found in ENCODE blacklist sites, and percentage of reads associated with TSS. D. Nuclei with <500 UMIs, >25,000 UMIs, and/or >5% mitochondrial reads were excluded from the snRNA-seq samples. E. There was a strong correlation of average gene expression between control and *Pitx2* mutant and between LA and PV, suggesting differences in individual genes are biological in origin and not technical.

Figure S2

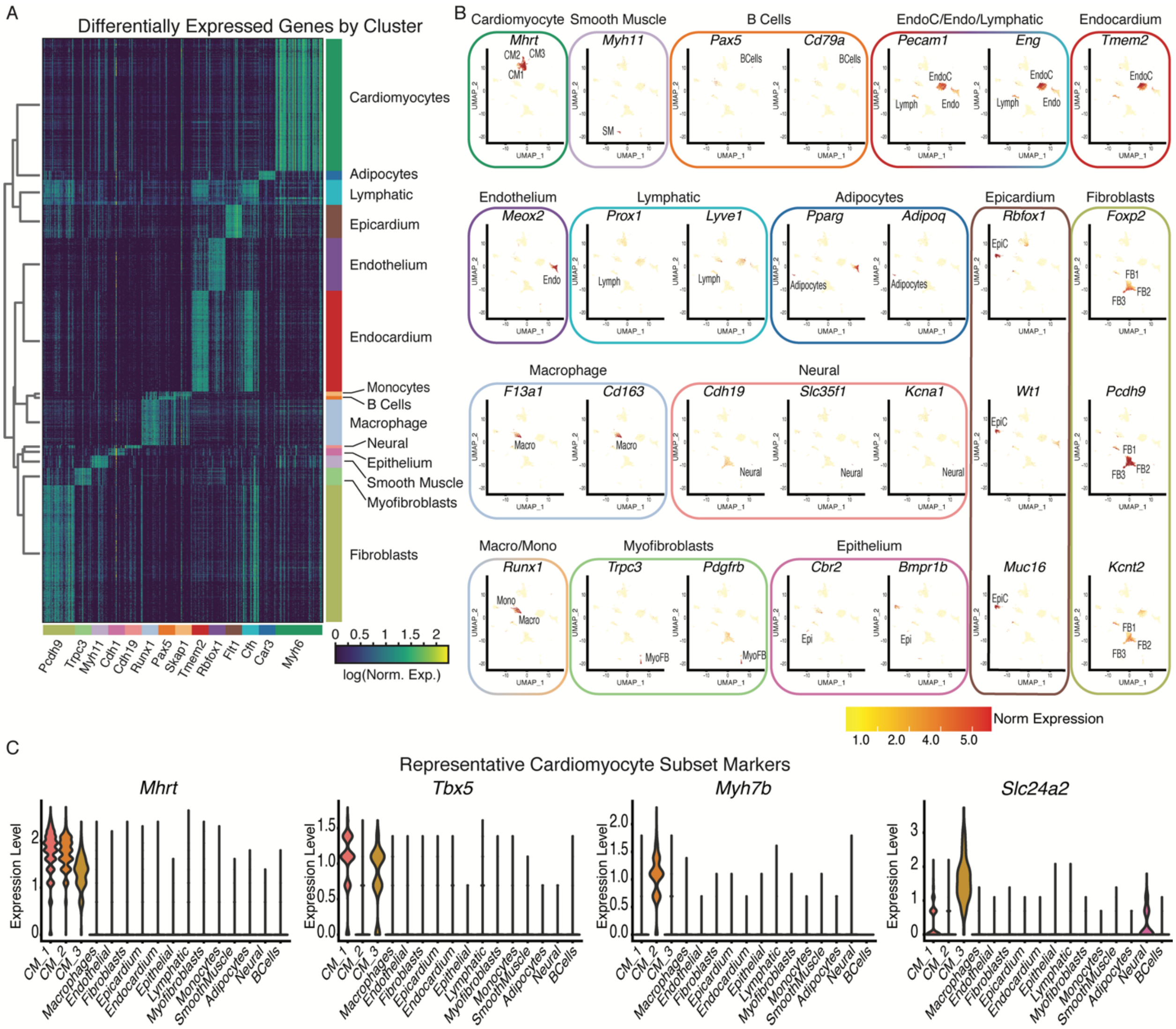

Figure S2. Cluster defining markers for snRNA-seq

A. Heatmap of top 20 differentially expressed genes for each cluster compared to all other clusters plotted in normalized expression in log-scale. Representative genes labeled on the x-axis. B. UMAP representation of the snRNA-seq data depicting the normalized gene expression for each marker gene utilized to call broad cell types. C. Violin plots depicting marker differences between the cardiomyocyte (CM) populations.

Figure S3

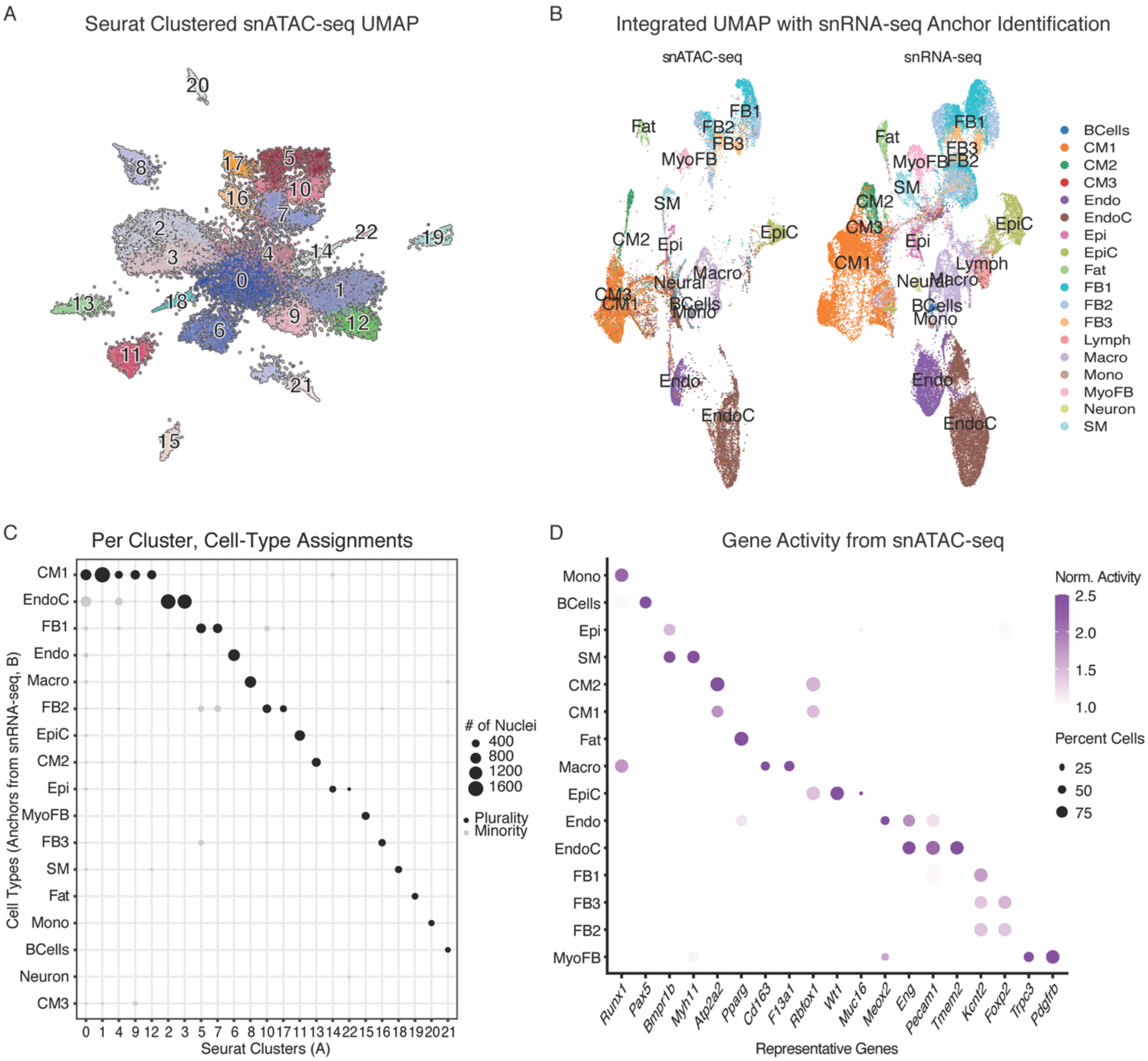

Figure S3. snATAC-seq clustering and label transfer

A. UMAP representation of the snATAC-seq data with all called clusters numbered. B. Integrated UMAP representation of the snRNA- and snATAC-seq datasets. Labels are derived from the transfer from snRNA-seq nuclei to snATAC-seq nuclei. C. Dot plot depicting the proportion of nuclei per cluster with cell type following label transfer from snRNA-seq dataset. Cluster numbers match those in panel A. Dots representing the largest percentage of nuclei, or plurality, are labeled in black and that label was applied to the entire snATAC-seq cluster from panel A. D. Normalized number of reads near the TSS, i.e., normalized gene activity, of representative cell type genes.

Figure S4

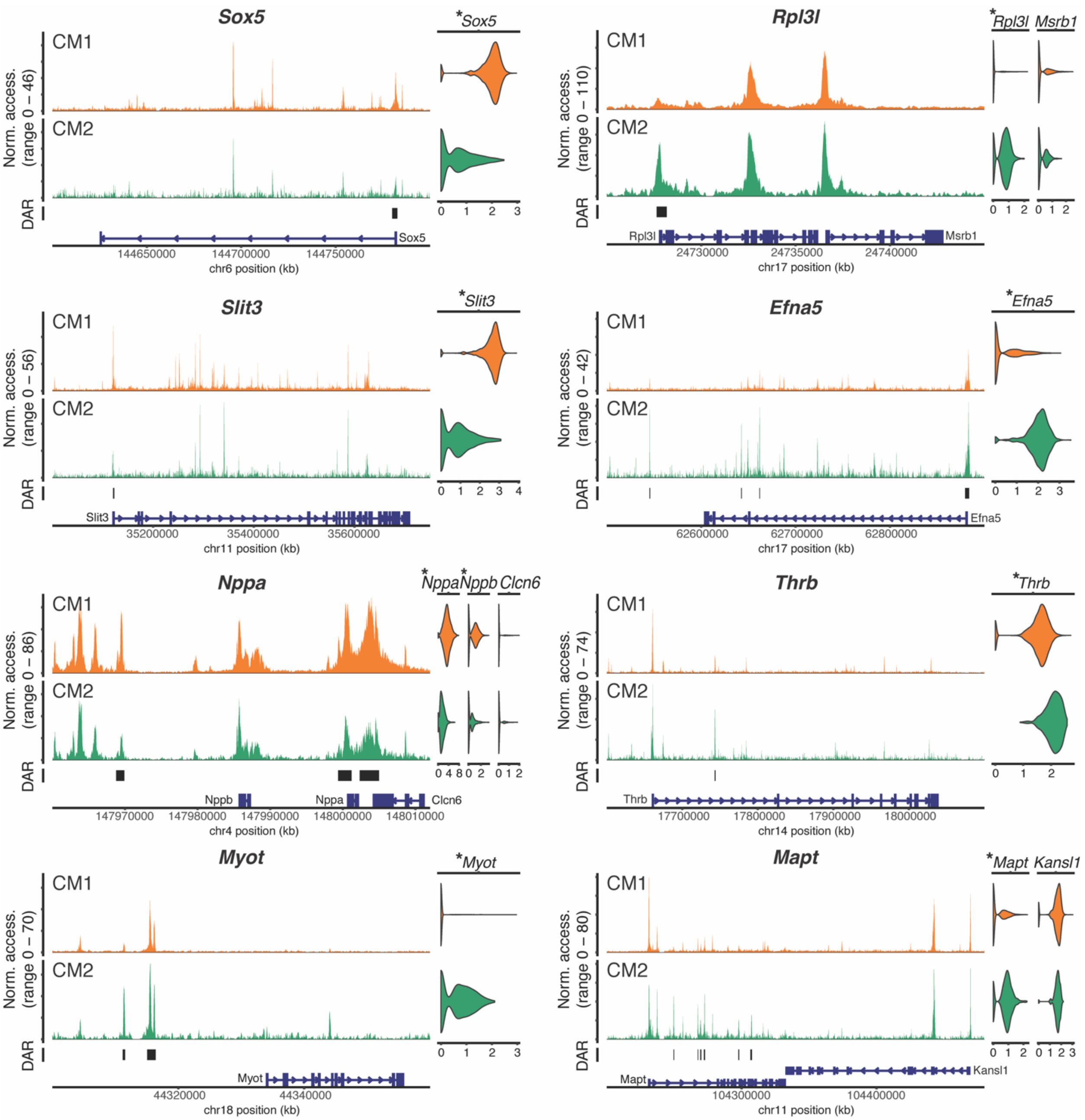

Figure S4. Examples of CM-subset enriched genes with differentially accessible regions

Genome browser views for additional differentially expressed genes (CM1 vs. CM2) associated with differentially accessible regions (DARs; CM1 vs. CM2). Violin plots to the right of the browser view show normalized gene expression from snRNA-seq for all genes in the viewpoint with the differentially expressed genes denoted (*).

Figure S5

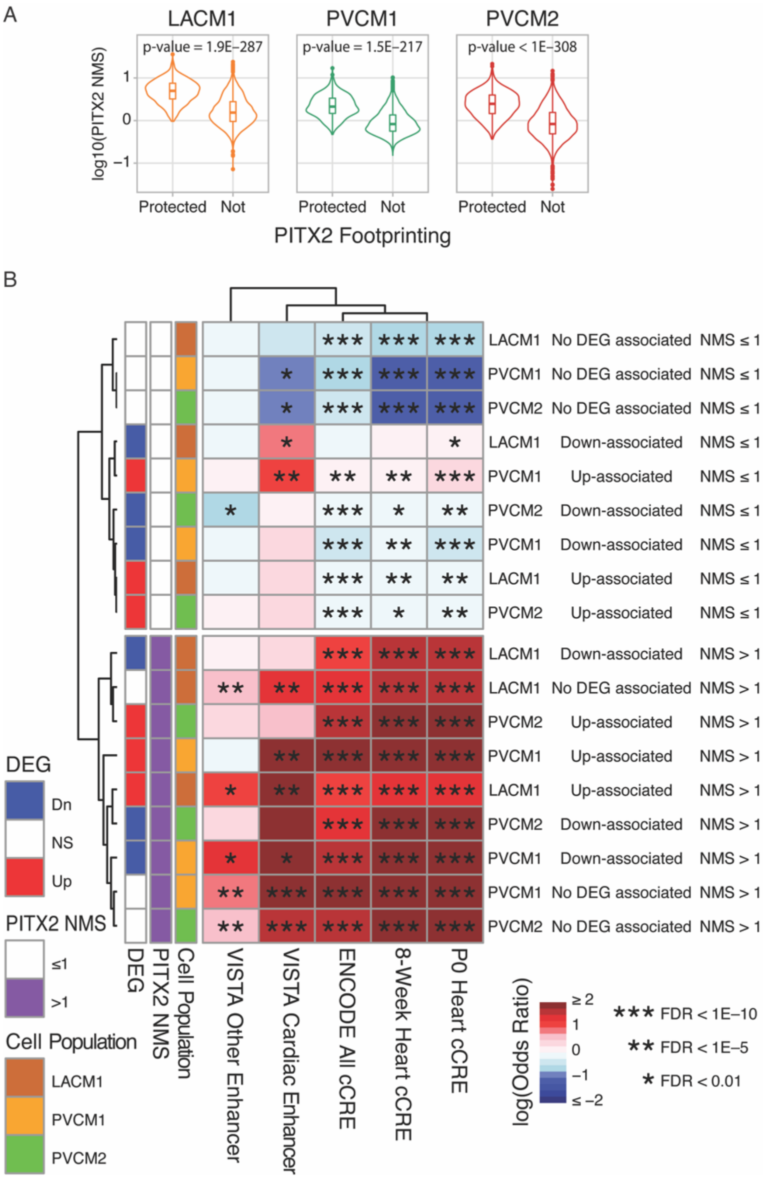

Figure S5. Functional relevance of PITX2 Sites in CM populations

A. Violin plots depicting the log10 PITX2 normalized motif score (NMS) calculated for LA CM1, PV CM1, and PV CM2 at sites with and without protected PITX2 motifs in the snATAC-seq data, i.e. PITX2 footprints. B. Heatmap of log Odds Ratio (Fisher's Exact Test, two-tailed) examining the enrichment or disenrichment of chromatin accessibility sites identified in CMs with the ENCODE and VISTA databases of validated and predicted CREs. Sites were broken up into whether they are associated with a DEG (Dn in blue = down-regulated in Pitx2 mutant, Up in red

= up-regulated in Pitx2 mutant, or NS in white = no DEG associated), contain a strong PITX2 site (PITX2 NMS > 1 in purple or PITX2 NMS ≤ 1 in white), and tissue source (LA CM1 in dark orange, PV CM1 in light orange, or PV CM2 in green). Asterisks refer to FDR < 1E–10 (***), FDR < 1E–5 (**), and FDR < 0.01 (*).

Figure S6

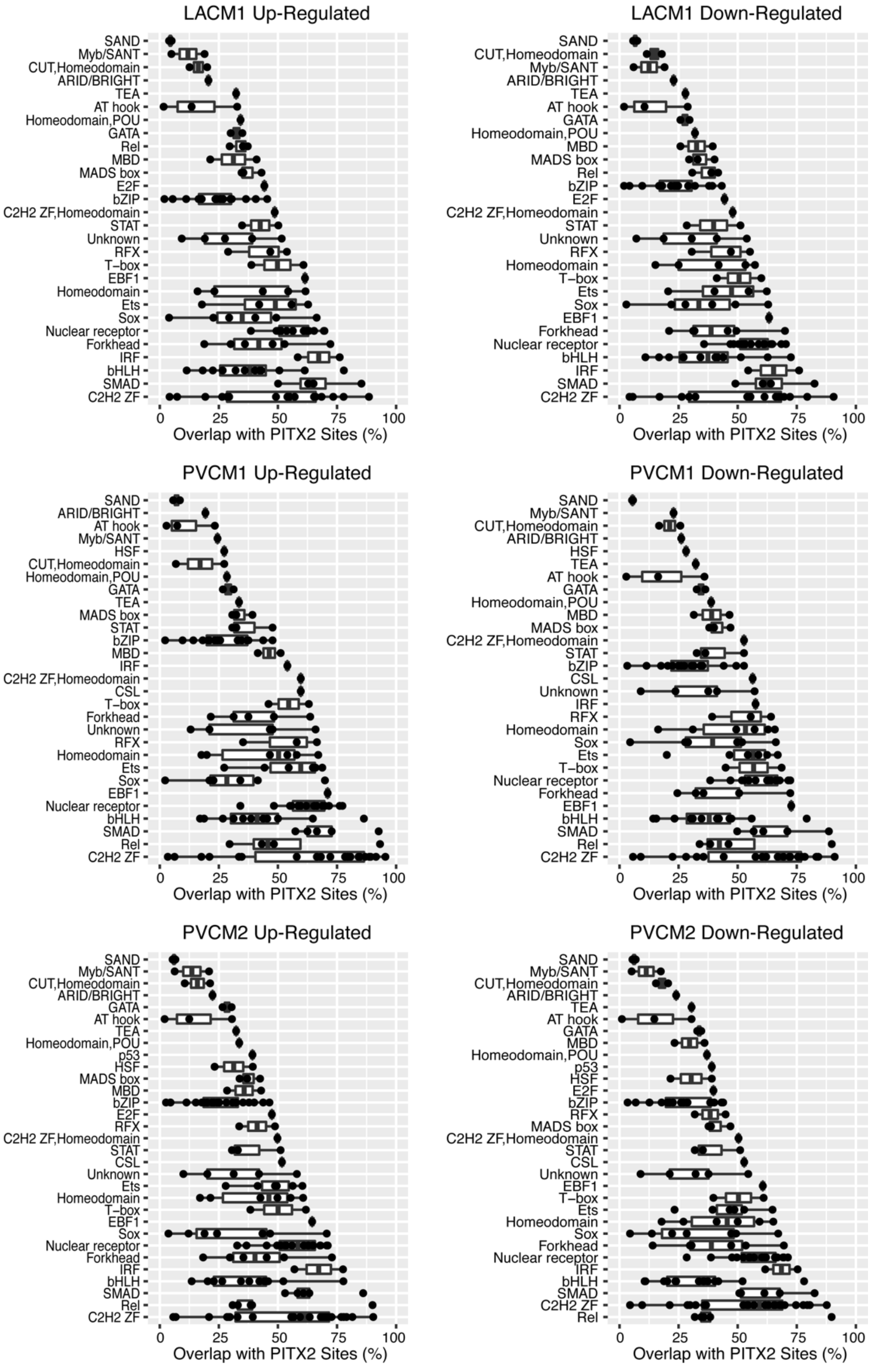
LACM1 Up-Regulated
SAND
Myb/SANT
CUT,Homeodomain
ARID/BRIGHT
TEA
AT hook
Homeodomain,POU
GATA
Rel
MBD
MADS box
E2F
bZIP
C2H2 ZF,Homeodomain
STAT
Unknown
RFX
T−box
EBF1
Homeodomain
Ets
Sox
Nuclear receptor
Forkhead
IRF
bHLH
SMAD
C2H2 ZF
0 25 50 75 100
Overlap with PITX2 Sites (%)
LACM1 Down-Regulated
SAND
CUT,Homeodomain
Myb/SANT
ARID/BRIGHT
TEA
AT hook
GATA
Homeodomain,POU
MBD
MADS box
Rel
bZIP
E2F
C2H2 ZF,Homeodomain
STAT
Unknown
RFX
Homeodomain
T−box
Ets
Sox
EBF1
Forkhead
Nuclear receptor
bHLH
IRF
SMAD
C2H2 ZF
0 25 50 75 100
Overlap with PITX2 Sites (%)
PVCM1 Up-Regulated
SAND
ARID/BRIGHT
AT hook
Myb/SANT
HSF
CUT,Homeodomain
Homeodomain,POU
GATA
TEA
MADS box
STAT
bZIP
MBD
IRF
C2H2 ZF,Homeodomain
CSL
T−box
Forkhead
Unknown
RFX
Homeodomain
Ets
Sox
EBF1
Nuclear receptor
bHLH
SMAD
Rel
C2H2 ZF
0 25 50 75 100
Overlap with PITX2 Sites (%)
PVCM1 Down-Regulated
SAND
Myb/SANT
CUT,Homeodomain
ARID/BRIGHT
HSF
TEA
AT hook
GATA
Homeodomain,POU
MBD
MADS box
C2H2 ZF,Homeodomain
STAT
bZIP
CSL
Unknown
IRF
RFX
Homeodomain
Sox
Ets
T−box
Nuclear receptor
Forkhead
EBF1
bHLH
SMAD
Rel
C2H2 ZF
0 25 50 75 100
Overlap with PITX2 Sites (%)
PVCM2 Up-Regulated
SAND
Myb/SANT
CUT,Homeodomain
ARID/BRIGHT
GATA
AT hook
TEA
Homeodomain,POU
p53
HSF
MADS box
MBD
bZIP
E2F
RFX
C2H2 ZF,Homeodomain
STAT
CSL
Unknown
Ets
Homeodomain
T−box
EBF1
Sox
Nuclear receptor
Forkhead
IRF
bHLH
SMAD
Rel
C2H2 ZF
0 25 50 75 100
Overlap with PITX2 Sites (%)
PVCM2 Down-Regulated
SAND
Myb/SANT
CUT,Homeodomain
ARID/BRIGHT
TEA
AT hook
GATA
MBD
Homeodomain,POU
p53
HSF
E2F
bZIP
RFX
MADS box
C2H2 ZF,Homeodomain
STAT
CSL
Unknown
EBF1
T−box
Ets
Homeodomain
Sox
Forkhead
Nuclear receptor
IRF
bHLH
SMAD
C2H2 ZF
Rel
0 25 50 75 100
Overlap with PITX2 Sites (%)

Figure S6. Percent PITX2 co-occurrence by comparison

Motif scanning was performed on all PITX2-containing cis-regulatory elements (CREs) associated with up- (left) or down-regulated (right) genes in LA CM1, PV CM1, or PV CM2. All motifs present in the CisBP database and expressed in the given cell type were scanned and grouped by motif family (y-axis).

Figure S7

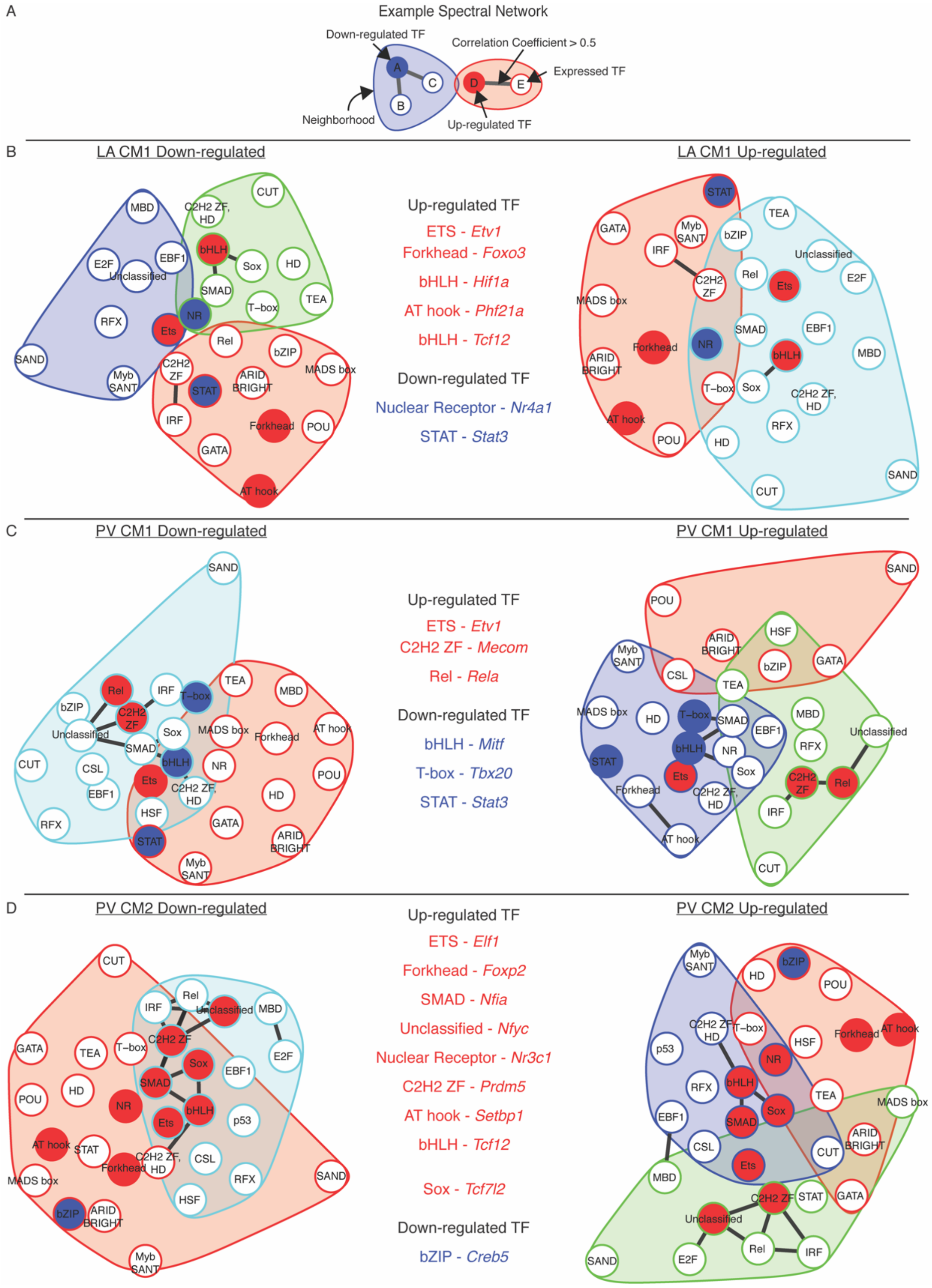
A
Example Spectral Network
Down-regulated TF
Correlation Coefficient > 0.5
Expressed TF
Neighborhood
Up-regulated TF
B
LA CM1 Down-regulated
LA CM1 Up-regulated
Up-regulated TF
ETS - Etv1
Forkhead - Foxo3
bHLH - Hif1a
AT hook - Phf21a
bHLH - Tcf12
Down-regulated TF
Nuclear Receptor - Nr4a1
STAT - Stat3
C
PV CM1 Down-regulated
PV CM1 Up-regulated
Up-regulated TF
ETS - Etv1
C2H2 ZF - Mecom
Rel - Rela
Down-regulated TF
bHLH - Mitf
T-box - Tbx20
STAT - Stat3
D
PV CM2 Down-regulated
PV CM2 Up-regulated
Up-regulated TF
ETS - Elf1
Forkhead - Foxp2
SMAD - Nfia
Unclassified - Nfyc
Nuclear Receptor - Nr3c1
C2H2 ZF - Prdm5
AT hook - Setbp1
bHLH - Tcf12
Sox - Tcf7l2
Down-regulated TF
bZIP - Creb5

Figure S7. Spectral clustering analysis for PITX2 co-localizing motifs

A. Co-localized motifs at PITX2-containing cis-regulatory elements (CREs) associated with DEGs were correlated with each other, and the matrix of correlations were provided as the input for spectral clustering. Spectral analyses were performed by transcription factor (TF) family. TF families with a similar network of associations are connected by colored “neighborhoods” of interaction (red, blue, green circles), which demonstrate higher cross-connectivity of the nodes within a neighborhood than between neighborhoods. On the other hand, strong, direct correlations (correlation coefficients > 0.5) are connected by solid gray lines. TF families containing an up- or down-regulated member are colored red or blue, respectively. B-D. Spectral clustering networks for each cardiomyocyte cell type, LA CM1 (B), PV CM1 (C), and PV CM2 (D), split by PITX2-containing CREs at down- and up-regulated genes. TFs that are differentially expressed are highlighted in the center for each comparison and colored red (up) or blue (down).

Figure S8

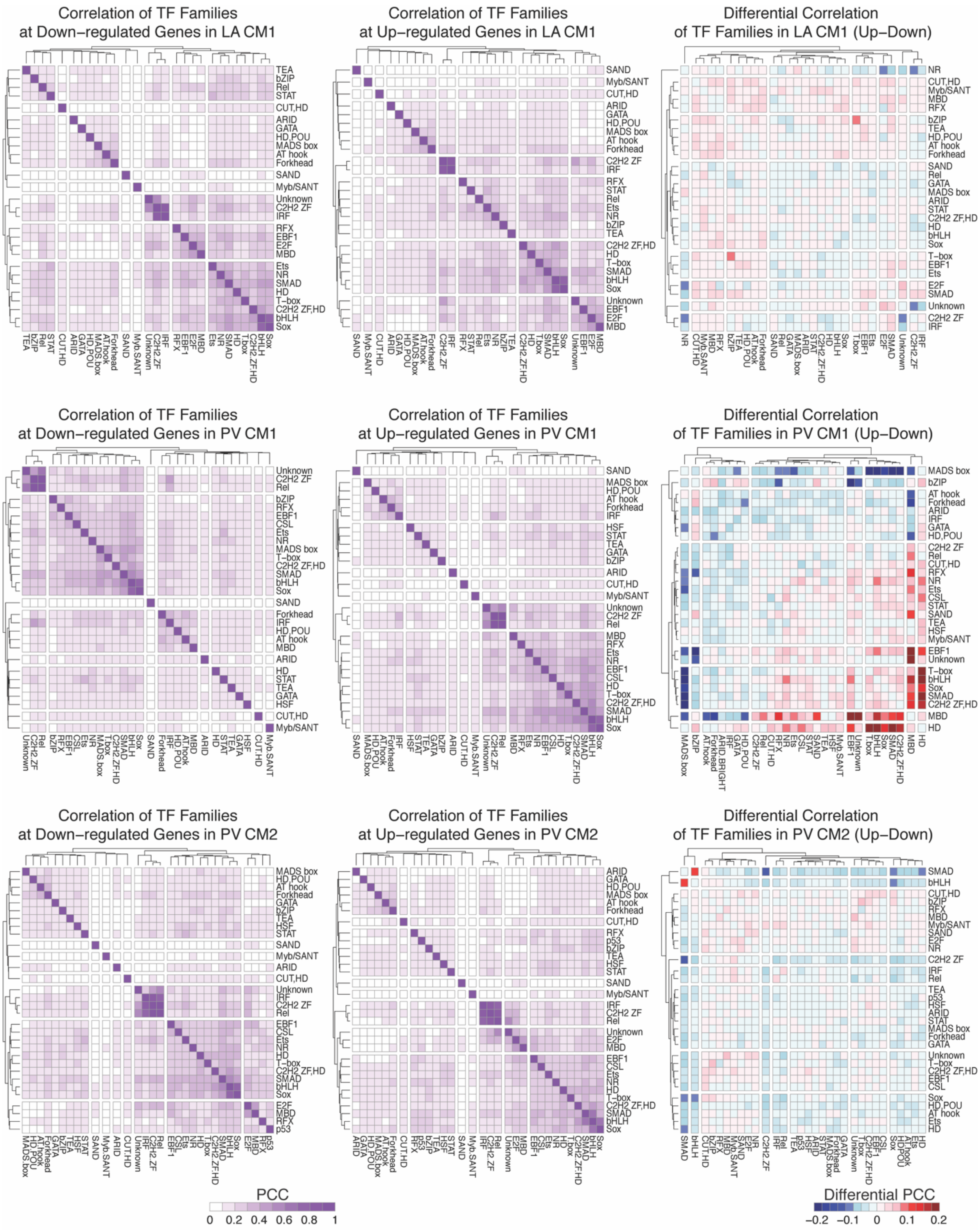

Correlation of TF Families at Down−regulated Genes in LA CM1
Correlation of TF Families at Up−regulated Genes in LA CM1
Differential Correlation of TF Families in LA CM1 (Up−Down)
Correlation of TF Families at Down−regulated Genes in PV CM1
Correlation of TF Families at Up−regulated Genes in PV CM1
Differential Correlation of TF Families in PV CM1 (Up−Down)
Correlation of TF Families at Down−regulated Genes in PV CM2
Correlation of TF Families at Up−regulated Genes in PV CM2
Differential Correlation of TF Families in PV CM2 (Up−Down)
PCC
0 0.2 0.4 0.6 0.8 1
Differential PCC
−0.2 −0.1 0 0.1 0.2

Figure S8. Differential correlation of co-localizing motifs

Pearson correlations were calculated across each motif family to examine the co-localization of motifs within cis-regulatory elements (CREs) at differentially expressed genes (DEGs). Correlation heatmaps associated with down-regulated genes are presented in the left column and up-regulated genes in the middle column. The differential correlations (Up-Down) are presented in the third column, such that TF families sharing a stronger correlation at up-regulated genes are positive while TF families sharing a strong correlation at down-regulated genes are negative. This analysis was performed for each LA CM1 (top row), PV CM1 (middle row), and PV CM2 (bottom row).

Figure S9

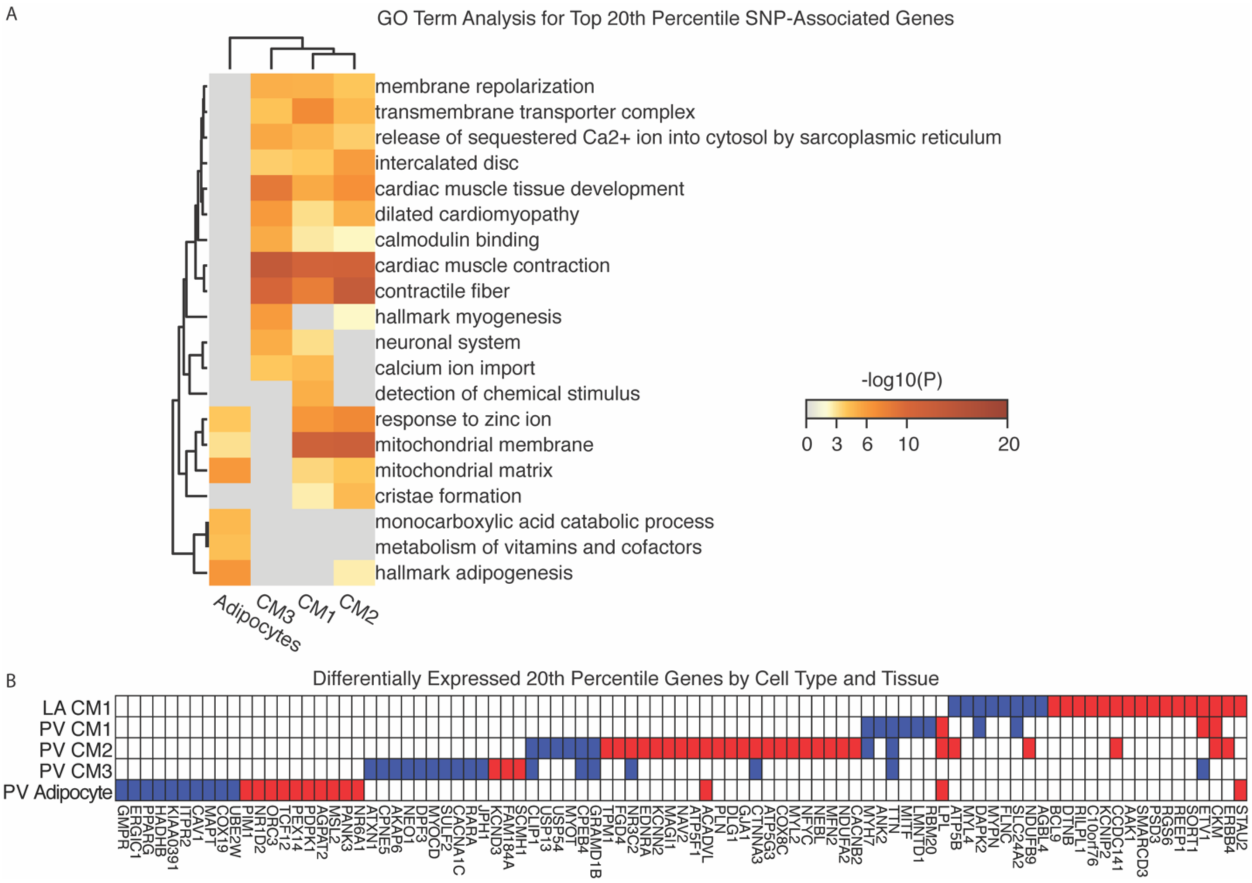

Figure S9. Examination of AF SNP-associated cardiomyocyte and adipocyte genes

A. Top parent GO terms identified for the AF SNP-associated genes from each CM1, CM2, CM3, and the adipocytes. B. List of all *Pitx2* DEGs associated with an AF GWAS locus for each the LA CM1, PV CM1, PV CM2, PV CM3, and PV Adipocytes. Genes up-regulated in the *Pitx2* mutant are indicated by a red box, while genes down-regulated in the *Pitx2* mutant are indicated by a blue box.

Figure S10

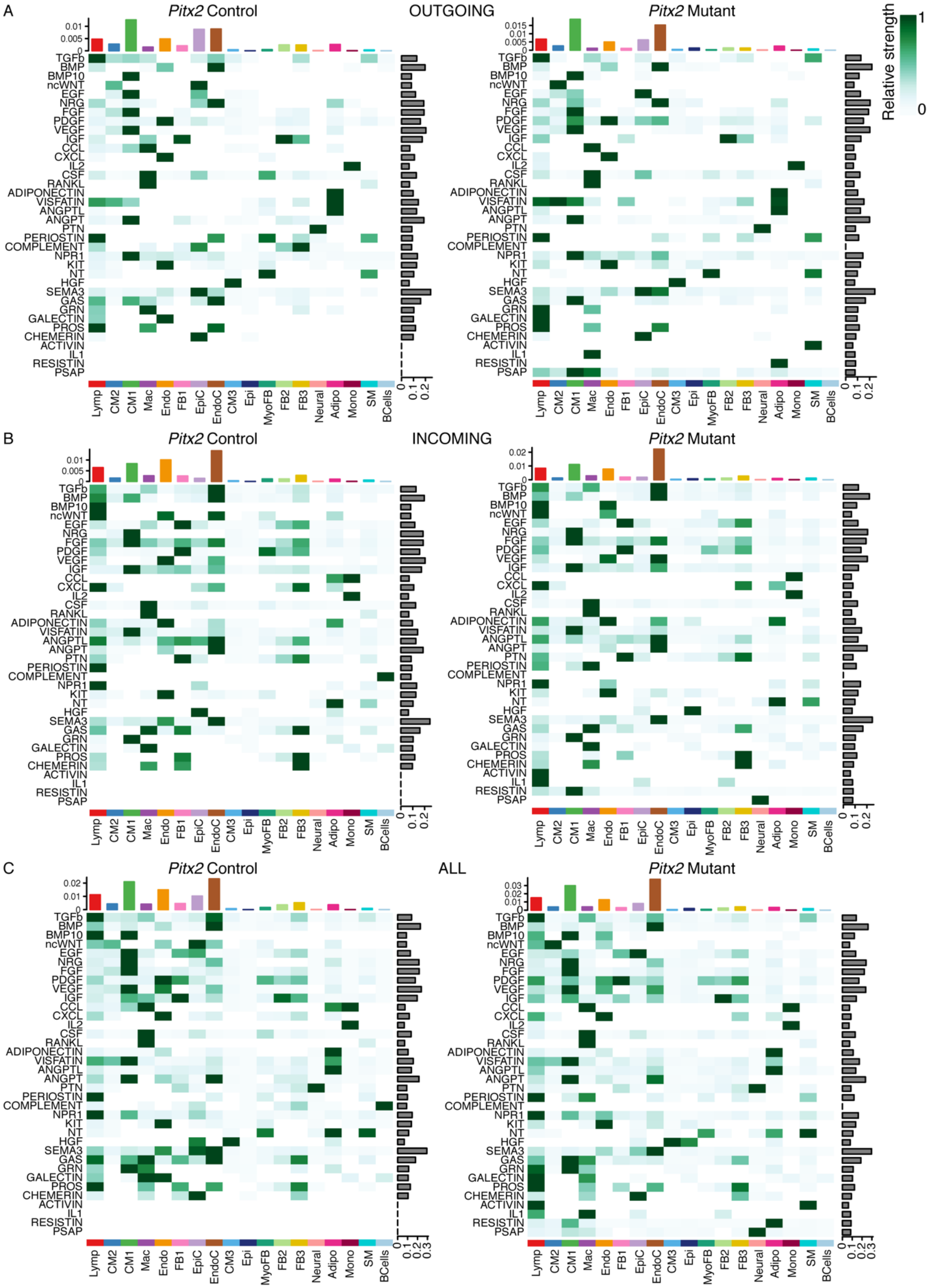
A
Pitx2 Control
OUTGOING
Pitx2 Mutant
B
Pitx2 Control
INCOMING
Pitx2 Mutant
C
Pitx2 Control
ALL
Pitx2 Mutant
Relative strength
0
1
TGFb
BMP
BMP10
ncWNT
EGF
NRG
FGF
PDGF
VEGF
IGF
CCL
CXCL
IL2
CSF
RANKL
ADIPONECTIN
VISFATIN
ANGPTL
ANGPT
PTN
PERIOSTIN
COMPLEMENT
NPR1
KIT
NT
HGF
SEMA3
GAS
GRN
GALECTIN
PROS
CHEMERIN
ACTIVIN
IL1
RESISTIN
PSAP
Lymp
CM2
CM1
Mac
Endo
FB1
EpiC
EndoC
CM3
Epi
MyoFB
FB2
FB3
Neural
Adipo
Mono
SM
BCells

Figure S10. Complete cell-cell communication for control and *Pitx2* mutant nuclei

Complete outputs generated by CellChat for outgoing (A), incoming (B), and outgoing/incoming (ALL, C) split by control (left) and *Pitx2* mutant (right).

SUPPLEMENTARY TABLE LEGENDS

Table S1. Cluster enriched gene expression

Gene expression was compared between the given cluster and all other clusters. Top 20 genes were reported per cluster.

Table S2. Pairwise comparison of CM subset gene expression

Significant gene expression differences compared between CM1 and CM2 (tab CM1vs.CM2”), CM1 and CM3 (tab CM1vs.CM3), and CM2 and CM3 (tab CM2vs.CM3). Differentially expressed genes (DEGs) are those with an FDR < 0.01 and |logFC| > 0.25.

Table S3. GO terms for CM subsets

The first tab is the complete list of GO terms identified for each cluster with identified genes (GO_AllLists). The second tab is the gene annotation for genes in comparison (Gene_Annotation). The third tab is parent and child terms for the most significantly enriched terms across all three CM subsets (AllCM_Enrichment).

Table S4. DARs identified between CM1 and CM2

The first tab lists all DARs more accessible in CM1 than CM2 and the DEGs are those that are more highly expressed in CM1 than CM2. The second tab lists all DARs more accessible in CM2 than CM1 and the DEGs are those that are more highly expressed in CM2 than CM1. AF GWAS are those same genes identified in GWAS Catalog.

Table S5. DEGs identified comparing control and *Pitx2* mutant for a given CM cluster

The different tabs have complete tables for the DEGs identified in each subset, i.e., LA CM1, PV CM1, PV CM2, and PV CM3 control vs. *Pitx2* mutant comparison. Up and down refers to up- and down-regulated genes in the *Pitx2* mutant condition, respectively.

Table S6. *Pitx2*-dependent genes identified in both LA and PV CM1

Table is split into those that were up-regulated in both comparisons and those that were down-regulated in both comparisons. Literature review of known functions and relationship to AF or cardiac diseases.

Table S7. The PITX2-containing CREs associated with DEGs

This Excel book is laid out by tissue and cell-type comparison, i.e., LA or PV, CM1 or CM2. Up-regulation (Up) or down-regulation (Dn) is in reference to *Pitx2* mutant condition. In the tables, Signal refers to the normalized signal under the peak for the given cell type (control and *Pitx2* mutant combined). The NMS is calculated as described in the Methods. Gene refers to the DEG identified. Note: Some PITX2-containing CREs are associated with more than one DEG but is only utilized one time in downstream analyses.

Table S8. DEGs identified comparing control and *Pitx2* mutant for endothelium and endocardium

Complete list of significantly up- and down-regulated genes in the *Pitx2* mutant condition for the endothelial (Endo) and endocardial (EndoC) clusters. The different tabs have complete tables for the DEGs identified in a given subset, i.e., Endo and EndoC control vs. *Pitx2* mutant comparison.

Table S9. GO terms for *Pitx2*-dependent endothelial and endocardial genes

The first tab is the complete list of GO terms identified for each cluster with identified genes (GO_AllLists). The second tab is the gene annotation for genes in comparison (Gene_Annotation). The third tab is parent and child terms for the most significantly enriched terms across all four Endo/EndoC lists (AllCM_Enrichment).
